# CherryPicker: An Algorithm for the Automated Parametrization of Large Biomolecules for Molecular Simulation

**DOI:** 10.3389/fchem.2019.00400

**Published:** 2019-06-05

**Authors:** Ivan D. Welsh, Jane R. Allison

**Affiliations:** ^1^School of Biological Sciences, University of Auckland, Auckland, New Zealand; ^2^Centre for Theoretical Chemistry and Physics, Institute of Natural and Computational Sciences, Massey University, Auckland, New Zealand; ^3^Biomolecular Interaction Centre, University of Canterbury, Christchurch, New Zealand; ^4^Maurice Wilkins Centre for Molecular Biodiscovery, University of Auckland, Auckland, New Zealand

**Keywords:** automated parametrization, fragment matching, biomolecules, molecular dynamics, graph theory, GROMOS force field

## Abstract

Molecular simulations allow investigation of the structure, dynamics and thermodynamics of molecules at an atomic level of detail, and as such, are becoming increasingly important across many areas of science. As the range of applications increases, so does the variety of molecules. Simulation of a new type of molecule requires generation of parameters that result in accurate representation of the behavior of that molecule, and, in most cases, are compatible with existing parameter sets. While many automated parametrization methods exist, they are in general not well suited to large and conformationally dynamic molecules. We present here a method for automated assignment of parameters for large, novel biomolecules, and demonstrate its usage for peptides of varying degrees of complexity. Our method uses a graph theoretic representation to facilitate matching of the target molecule to molecular fragments for which reliable parameters are available. It requires minimal user input and creates parameter files compatible with the widely-used GROMACS simulation software.

## 1. Introduction

Molecular simulations provide a means to investigate molecular interactions at or near atomistic detail. Such simulations are becoming increasingly important in areas ranging from biomedical to materials science. The accuracy of molecular simulations is in large part dependent on the quality of the parameters used to describe the inter- and intra- molecular interactions; that is, the force field. Within the realm of biomolecular simulations, force fields such as AMBER (Weiner et al., 1984, 1986; Cornell et al., 1995; Duan et al., 2003), CHARMM (Brooks et al., 1983; Reiher, 1985; MacKerell et al., 1998, 2000; Foloppe and MacKerell, 2000) and GROMOS (Schuler et al., 2001; Oostenbrink et al., 2004; Poger et al., 2010; Schmid et al., 2011) are widely used. These contain highly optimized parameters for proteins, and to varying degrees, nucleic acids, lipids and sugars. However, when it comes to simulating novel molecules, such as drug molecules, toxins, non-ribosomal peptides, post-translational modifications to proteins, or certain lipids, parameters are unlikely to be included in a given force field, necessitating parametrization.

Over the decades, a range of automated parametrization methods have been developed, such as MKTOP (Kaminski et al., 2001; Ribeiro et al., 2008), Antechamber (Wang et al., 2004, 2006), PRODRG (Schüttelkopf and van Aalten, 2004), the ATB (Malde et al., 2011; Canzar et al., 2013; Koziara et al., 2014), Paramfit (Betz and Walker, 2015), GENRTF (Miller et al., 2008), RED (Vanquelef et al., 2011) and LigParGen (Dodda et al., 2017). Such methods generally rely on performing quantum chemical calculations and deriving atomic partial charges and bonded parameters from the resultant electronic information.

As the size of the molecule to be parameterized increases, such quantum chemical based methods become impractical. Firstly, quantum chemical methods scale at least as poorly as *N*^3^, where *N* is the number of basis functions, with many scaling even more poorly. As such, the required computational effort quickly becomes intractable as molecule size increases. Additionally, it is well known that charge distributions are highly conformationally dependent (Stouch and Williams, 1992, 1993; Urban and Famini, 1993; Koch and Stone, 1996). As a consequence, derivation of reliable force field parameters capable of describing the full conformational space of the molecule requires quantum chemical calculations to be carried out for multiple conformations. This conformational dependence applies to both non-bonded and, to a lesser extent, bonded parameters.

Historically, biomolecule force fields were constructed by careful parametrization of small model chemicals, for example, compounds representative of each amino acid side chain. A similar approach could conceivably be taken to generate parameters for novel molecules. Proteins have a very limited chemical space, comprising just 20 unique amino acids that are joined together in a linear fashion, and which themselves mostly contain an identical “backbone.” In comparison, the chemical space available to drug molecules is extremely large. For example, the ZINC15 database (Sterling and Irwin, 2015) contains over 100 million unique molecules. This greatly increased chemical space makes it implausible to define a finite set of model compounds. However drug molecules are typically relatively small, and are often conformationally constrained, making automated parameterisation schemes both tractable and best suited to their parameterisation. Biomolecules besides proteins and other well-studied cases present a different scenario, however. Their chemical space is much more limited, but their size can extend well beyond the reaches of quantum chemical calculations. Additionally, they are likely to undergo significant conformational motion.

We have therefore developed a fragment-based approach, reminiscent of the construction of biomolecular force fields, to parameterisation of novel biomolecules. Our approach utilizes graph theoretic representation and methodology. In contrast to other existing parameterisation methods, our approach avoids the use of expensive quantum chemical calculations. Our implementation is split into two parts: an Athenaeum, a library of molecular fragments, and CherryPicker, an algorithm for parameterisation based on matching portions of a target molecule to fragments in an Athenaeum. The code is written in C++, with Python bindings to enable ease of use provided by pybind11 (Jakob et al., 2017), and is available on GitHub at https://git.io/fp4Fr.

## 2. Algorithm Description

**Figure 1:**
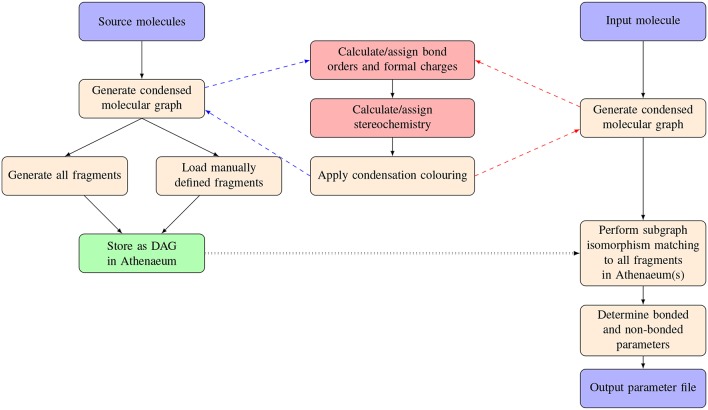
Schematic of the process of automated parameterisation carried out by CherryPicker. The two red boxes indicate procedures described elsewhere (Welsh and Allison, 2019).

An overview of the procedures carried out in parameterizing a novel molecule using CherryPicker and one or more Athenaeums is provided in Figure 1. Parameterizing a novel molecule using CherryPicker proceeds as follows. A target molecule is submitted to CherryPicker and parameters are determined by comparing the target with molecular fragments contained in one or more Athenaeums. We use a graph theoretic framework in which molecules are represented as condensed molecular graphs, and matching between the target molecule and the fragments in the Athenaeum is done using subgraph isomorphism. A default parameterisation scheme is provided where parameters assigned to the target molecule are the mean (point charges) or mode (all other parameters) of the set of parameter values obtained from the pool of matching fragments. The set of assigned parameters is written to file in a format suitable for use in a simulation engine such as GROMACS (Abraham et al., 2015).

Below, we describe the implementation details of the Athenaeum library and CherryPicker algorithm. For clarity, we assume the use of the GROMOS force field and the GROMACS simulation engine. This entails the parameter values for each force field term, such as the parameters of the Lennard-Jones term for atomic centers or harmonic force constants for bonds, being identified by an integer type code. The only exception is the atomic partial charges, for which the actual parameter value is used throughout. The implementation is designed such that adding support for additional force fields or simulation engines is straightforward, however.

### 2.1. Data Input and Output

We provide a number of file input and output methods for handling chemical structure and parameter data.

To build an Athenaeum, both the chemical structure and the associated parameters of one or more molecules are required. Supported input file formats are the MTB format used by the GROMOS simulation engine (Christen et al., 2005), and the ITP format used by GROMACS (Abraham et al., 2015). It is also possible to supply a PDB file alongside the MTB or IFP file. ITP parsing currently assumes a GROMOS-style force field. As MTB and, in many cases, ITP files use force field type codes rather than specifying the actual parameter values, the force field itself, i.e., the parameter values associated with each force field type code, is also required. Currently only the GROMOS IFP file format is able to be parsed, but a hard-coded implementation of the GROMOS 54A7 force field (Schmid et al., 2011) is also provided.

The chemical structure of the target molecule for parameterisation must be provided in PDB format, including CONECT records. Chemical structures can be output in either PDB or GROMACS GRO formats, and the associated assigned parameters can be output in MTB or IFP format.

### 2.2. Condensed Molecular Graph

The target molecule and all fragments in the Athenaeum are represented as *molecular graphs* or *condensed molecular graphs*. A *molecular graph* is a labeled graph whose vertices correspond to the atoms of a molecule and edges correspond to the chemical bonds. Generally, vertices are labeled with the element type of the atom, and edges are labeled with the type of the bond, i.e., its bond order. Such graphs often contain a large number of leaves, which can cause combinatorial explosion of search algorithms. This is particularly true of the subgraph isomorphism mappings used here. To alleviate this problem we introduce the concept of a *condensed molecular graph*.

A condensed molecular graph is a molecular graph where leaves are removed and the label of the leaf's parent vertex modified to indicate the absence. A leaf is only removed if its corresponding atom has a formal charge of 0, is either hydrogen or a halogen, and the edge that would be removed is labeled with a bond order of one. In this way, we ensure that any potentially important chemical information is explicitly maintained while reducing the computational cost.

Another reason for the use of condensed molecular graphs is their increased information density. Bit manipulation is utilized to compress multiple pieces of information into a single integer value for labeling both the vertices and edges. Doing so means that when performing subgraph isomorphism matching, the comparison between two potentially matching vertices or edges can be executed in a single CPU instruction.

#### 2.2.1. Vertex Labels

**Figure 2:**
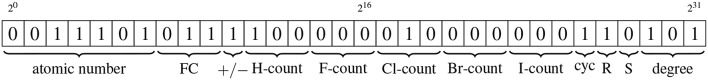
Schematic illustrating how bit manipulation allows all information associated with a vertex to be stored using just 32 bits. The types of information are, from left to right: atomic number of the element associated with the vertex; magnitude of the formal charge of the atom associated with the vertex; sign of the formal charge; the counts of each of the five different elements that can be condensed into a vertex; whether the vertex is part of a cycle of size up to eight; the chirality (R/S) of the vertex; the degree of the vertex. All items are stored by converting the positive numeric values to binary format, other than the sign of the formal charge and the cycle and chirality indicators, for which the binary encodings are described in the text.

The vertices of the condensed molecular graph carry ten distinct pieces of information. Bit manipulation means that each vertex label requires just 32 bits, as shown in Figure 2 and detailed below.

##### 2^0^ → 2^6^

These seven bits represent the atomic number, in binary form, of the element associated with the vertex. Currently, there are 118 elements in the periodic table, meaning they can all be represented by the seven available bits.

##### 2^7^ → 2^9^

These three bits give the magnitude of the formal charge on the atom associated with the vertex, again in binary form. The use of three bits allows for formal charge magnitudes of between zero and seven, which covers all formal charge values likely to occur in the context of molecular simulation. An automated means of determining formal charge, such as that previously described by us (Welsh and Allison, 2019), can be utilized.

##### 2^10^

This bit gives the sign of the formal charge. It is set to 0 if the formal charge is zero or positive and 1 if it is negative.

##### 2^11^ → 2^13^, 2^14^ → 2^16^, 2^17^ → 2^19^, 2^20^ → 2^22^, 2^23^ → 2^25^

These five groups of three bits represent the counts of each of the five different elements that could be condensed into a vertex in the transition from a molecular graph to a condensed molecular graph, which are limited to hydrogen or halogen atoms. While in most molecular contexts, no more than three vertices of the same element would be condensed into the same parent vertex, we allow three bits per element rather than just two so as to allow for cases such as methane where there are four condensed vertices. The value of each bit is the binary value of the integer count of that element, with the first group being for condensed hydrogen atoms, the second for condensed fluorine and so forth down the halogen column of the periodic table.

##### 2^26^

This bit is set if the vertex is part of a cycle of size up to eight.

##### 2^27^ → 2^28^

These two bits are used to represent any chirality associated with the atom represented by a vertex. Bit 2^27^ is set when the atom has *R* chirality, and bit 2^28^ is set when the atom has *S* chirality.

##### 2^29^ → 2^31^

These final three bits represent the degree, in binary representation, of the vertex within the molecular graph.

### 2.2.2. Edge Labels

**Figure 3:**
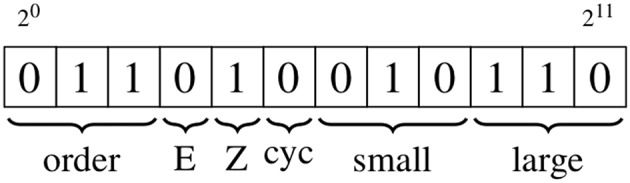
Schematic showing the edge information, which is stored using 12 bits. The types of information are, from left to right: bond order of the associated bond; stereochemistry (E/Z) of the associated bond; whether the edge is part of a cycle with a size of up to eight; the smallest and largest degree of the vertices on either side of the edge. The numeric values of the bond order and degree are converted to binary format; the binary encodings of the stereochemistry and cycle indicators are described in the text.

Edge labels utilize 12 bits to store four distinct pieces of information, again making use of bit manipulation, as shown in Figure 3 and detailed below.

#### 2^0^ → 2^2^

These three bits contain the bond order of the associated bond, with each value matching the integer value of the bond order, so that single to quadruple bonds carry values of 1−4, an aromatic bond has a value of 5, a one-and-a-half bond has a value of 6 and a two-and-a-half bond has a value of 7.

#### 2^3^ → 2^4^

These two bits are used to represent any stereochemistry associated with the bond represented by an edge. Bit 2^3^ is set when the bond is the central bond of an *E* isomer, and 2^4^ is set when the bond is the central bond of a *Z* isomer.

#### 2^5^

This bit is set if the edge is part of a cycle with a size of up to eight.

#### 2^6^ → 2^8^, 2^9^ → 2^11^

These two groups contain the degree of the vertices on either side of the edge, with the first group containing the lowest degree value, and the second the highest. The three bits in each group are set to match the binary value of the integer degree.

### 2.3. Athenaeum

The CherryPicker algorithm utilisers one or more Athenaeums, each of which is a collection of already-parameterized molecules and the fragments derived from them. Fragmentation can be user-directed or fully automated, as outlined below.

An Athenaeum can be marked as *self-consistent*. This label is intended for use in Athenaeums constructed from fragment sources, such as the amino acids, for which the parameter values assigned to a given functional group are consistent. Being labeled as such indicates that all matching fragments that the CherryPicker algorithm finds within the Athenaeum will carry the same parameter values. If any cases where this is not the case are discovered, an error will be thrown.

#### 2.3.1. Fragments

A fragment is a connected induced subgraph of a condensed molecular graph, consisting of two regions defined by non-intersecting vertex sets. The first region is the *core region*, from which parameters will be extracted. The vertex set of this region must also induce a connected subgraph of the overall condensed molecular graph. The second region is the *overlap region*. The overlap region does not contribute parameters; rather, it is used to ensure that the core region of a fragment and the portion of the target to which it matches have similar chemical environments. See the Supplementary Materials for a discussion on choosing the overlap size.

Fragments are stored within an Athenaeum in a tree like structure on a per molecule basis. A fragment is a vertex superset of another (smaller) fragment if the set of vertices in the smaller fragment are a subset of those in the larger fragment. Each fragment stores a reference to each of its immediate vertex supersets. However if multiple fragments are vertex subsets of another larger fragment, the larger fragment is only referenced by the first such smaller fragment.

#### 2.3.2. User-Specified Fragment Generation

User-specified fragment generation involves providing a molecule, and a list of the core and overlap atoms that will form a fragment. The fragment specification file format allows for the user to specify any number of fragments to generate from a given molecule. Examples are provided in the GitHub repository. The only restriction on user-specified fragments is that the core atoms and core + overlap atoms must form induced subgraphs of the molecule's condensed molecular graph. Unlike automatically generated fragments, the overlap regions of each fragment are specified individually, and may contain different numbers of atoms.

#### 2.3.3. Fully Automated Fragment Generation

For fully automated fragment generation, the only input required is a molecule. The overlap size is a property of the Athenaeum to which the fragments formed from this molecule will be added. Fragments of all possible sizes are generated. The user has the option to specify minimum and/or maximum fragment sizes, as well as choose which Athenaeum(s) to match against, when running CherryPicker.

For each provided molecule, all connected induced subgraphs *G*′ = (*V*′, *E*′) of the molecule's condensed molecular graph, *G* = (*V, E*), are generated and tested against the three criteria outlined below to determine whether they are valid fragments, with each subgraph forming the core region of a fragment. The overlap region is then all vertices of the subgraph *G*′ within a path length *k* of the core region, where *k* is the overlap length. Each Athenaeum generated fully automatically has a separate value of *k*.

The three criteria used to determine if a fragment is valid are:

1. Given a subgraph *G*′ = (*V*′, *E*′) of *G*, and edge *e* = (*u, v*) incident on *G*′, that is, (*u* ∈ *V* ∧ *v* ∈ *V* ′) ∨ (*u* ∈ *V* ′ ∧ *v* ∈ *V*), *e* must be labeled as representing a bond of order one or aromatic. Allowing aromatic bonds here enables fused cyclic systems to be fragmented.
2. Any edge *e* = (*u, v*) incident on *G*′ must have at least one of its vertices labeled as being carbon.
3. Let the subgraph *G*^′′^ = (*V*^′′^, *E*^′′^) be the induced subgraph of *G* containing the union of the core region vertex set and overlap region vertex set. Every leaf of *G*^′′^ that belongs to the overlap region vertex set must have a path length of at least *k* to all vertices of the core region.

The user can choose which of these rules to activate when generating an Athenaeum, and the implementation also makes it straightforward to add additional rules as desired.

### 2.4. CherryPicker

The CherryPicker algorithm assigns parameters to a target molecule by iterating through a list of Athenaeums. The user can determine the minimum and/or maximum size of the core region of the fragments to test, defaulting to a minimum size of four and no maximum size. At each iteration, all fragments within the current Athenaeum are checked for subgraph isomorphism with the target condensed molecular graph. If the fragment is found to match a portion of the target molecule, the values of the fragment's atom, bond, angle and dihedral parameters are tallied against the corresponding vertices and edges of the target condensed molecular graph. This process is repeated until all Athenaeums have been exhausted, or the target molecule has a parameter pool for all its terms. The resulting *target molecule parameter pool* is then returned.

CherryPicker allows the concept of *dangling* bonded parameters. This refers to the case where, when a fragment is matched to a target molecule, at least half of the atoms involved in a given bond, angle or dihedral term are within the core region of the fragment, and the remainder are in the overlap region. Parameters can be assigned from the dangling bonded term, effectively enabling the core region be to edge terminated rather than vertex terminated. The criteria for allowing a bonded term to dangle is that one of the two atoms defining a bond, two of the atoms defining an angle, or two neighboring atoms defining a dihedral must be in the core region.

Being NP-complete, the subgraph isomorphism problem is inherently difficult. A number of heuristic methods have been developed for solving this problem. Here, we have implemented and tested two algorithms for subgraph isomorphism: the VF2 algorithm (Cordella et al., 2004), as implemented in the Boost Graph Library (Siek et al., 2002), and the RI algorithm (Bonnici et al., 2013). The latter is the default setting as it is more efficient. Both algorithms allow for labeled vertices and edges of graphs. As the labels used here are single integer values, a *mask* is created prior to performing the subgraph isomorphism search that dictates the pieces of information in the label to consider. For example, vertex matches could be based upon only the element and formal charge components of the label without having to modify the label, and thus the Athenaeum, itself. Additionally, to further speed up the parameterisation time, we exploit the tree-like way in which fragments are stored in an Athenaeum. The smaller fragments are tested for subgraph isomorphism first. If a small fragment does not match the target molecule, all larger fragments of which the small fragment is a subgraph will also not match, and therefore do not need to be tested.

Athenaeums are searched in a first-in first-out manner. Ideally they are provided in an order running from most to least reliable. Once all fragments within an Athenaeum have been compared to the target molecule, all components of the target molecule which mapped to a fragment are marked. These regions are excluded from any subsequent searches through additional Athenaeums, thus ensuring that the most reliable parameters identified from the early-stage Athenaeums are not replaced by less reliable parameters from later Athenaeums. Additionally, if an Athenaeum is marked as self-consistent, once all fragments in that Athenaeum have been tested, the mapped parameters are checked to ensure that they are indeed self-consistent. If they are not, CherryPicker ceases execution and the user is alerted.

The target molecule parameter pool is a distinct representation of a molecule containing all parameter values mapped to it during the subgraph isomorphism searches through an Athenaeum. For atoms, the parameter pool consists of counts of the type of each mapped atom, and the list of partial atomic charges of each mapped atom. For bonds, angles and dihedrals the parameter pool consists of counts of the corresponding mapped types.

Once each Athenaeum has been searched, the target molecule parameter pool is distilled down to a set of *assigned parameter values* for the marked components of the target molecule. Partial charges for atoms are set to the mean of the distribution of mapped partial charges. Preliminary tests revealed that for fragments with an overlap size of at least two atoms, the partial charges are approximately normally distributed, thus the mean is appropriate (see Figures S1, S2). All other parameter values are set to the mode of the mapped types.

Once all Athenaeum's have been searched, the partial charges are checked. Adding the means of partial atomic charges from matched fragments is likely to result in a non-integer total charge on the target molecule. We therefore implement a simple scheme to avoid this physically unreasonable situation. We assume that the difference between the expected total charge of the target molecule and that obtained with CherryPicker is small. As such, the difference between the assigned and expected charge is added to the atom with the most negative (assigned charge > expected charge) or positive (assigned charge < expected charge) partial charge. Atoms whose parameters were determined by mapping to a self-consistent Athenaeum are excluded from charge adjustment.

CherryPicker outputs both the assigned parameter values and the entire molecule parameter pool. The latter given as comments so that the provided MTB or ITP files could be used without further modification. Providing the entire molecule parameter pool allows the user to check and, if desired, adjust the assigned parameters, as well as gain a deeper understanding of the performance of the CherryPicker algorithm and suitability of the Athenaeums.

## 3. Algorithm Testing

To illustrate the effectiveness of this fragment based method for parameterisation we present a few simple test molecules, focusing on the well studied peptide space. In future work we will explore much larger and more diverse chemical spaces.

**Figure 4:**
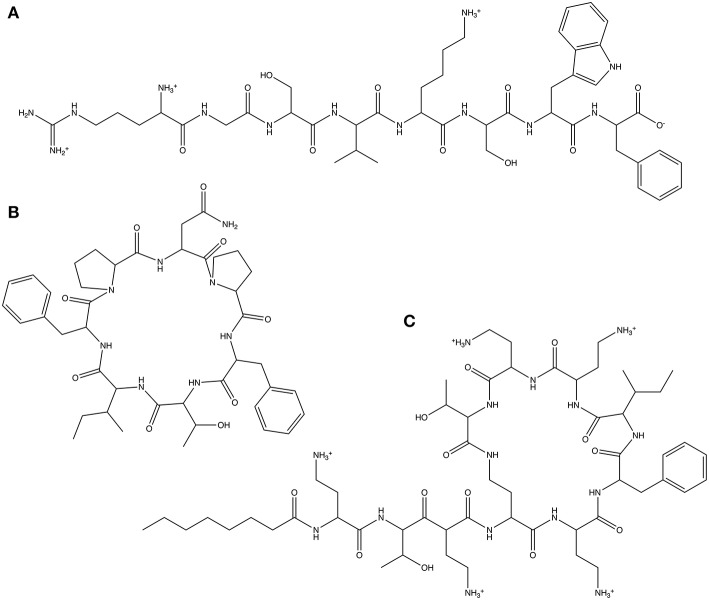
The three peptides used to illustrate the performance of CherryPicker using user-specified and automatically-generated Athenaeums. **(A)** The randomly generated amino acid sequence Arg-Gly-Ser-Val-Lys-Ser-Trp-Phe, **(B)** the cyclic peptide Axinellin A, which has the amino acid sequence cyclo(Asn-Pro-Phe-Thr-Ile-Phe-Pro), **(C)** the non-ribosomal cyclic lipopeptide Polymyxin B3, which has the amino acid sequence octanoyl-Dab-Thr-Dab-Dab(1)-Dab-d-Phe-Leu-Dab-Dab-Thr-(1).

As our first test, we use a linear octapeptide with the randomly generated amino acid sequence Arg-Gly-Ser-Val-Lys-Ser-Trp-Phe (Figure 4A). The second test molecule is the cyclic peptide *Axinellin A*, a bioactive cyclic heptapeptide isolated from the marine sponge *Axinella carteri* (Randazzo et al., 1998), which has been chemically synthesized (Fairweather et al., 2010) and has the amino acid sequence *cyclo*(Asn-Pro-Phe-Thr-Ile-Phe-Pro) (Figure 4B). Finally, we look at the non-ribosomal peptide *Polymyxin B3* (Zavascki et al., 2007), a lipopeptide antibiotic isolated from *Bacillus polymyxa*. Polymyxin B3 comprises a cyclic polypeptide with a tripeptide side chain and a fatty acid tail and has the amino acid sequence octanoyl-Dab-Thr-Dab-Dab(1)-Dab-D-Phe-Leu-Dab-Dab-Thr-(1) (Figure 4C). The parameters obtained for each test molecule using CherryPicker are provided in the Supplementary Material.

The CherryPicker algorithm was run with masked vertex and edge labels. Vertices were masked such that only the element type, formal charge, condensed vertices, and degree were used for matching. Edge labels were masked such that only bond order, and the source/target degree were used. For the automatically generated Athenaeums, the minimum fragment size, that is, the size of the core region in a fragment graph, was two.

### 3.1. Athenaeums

**Figure 5:**
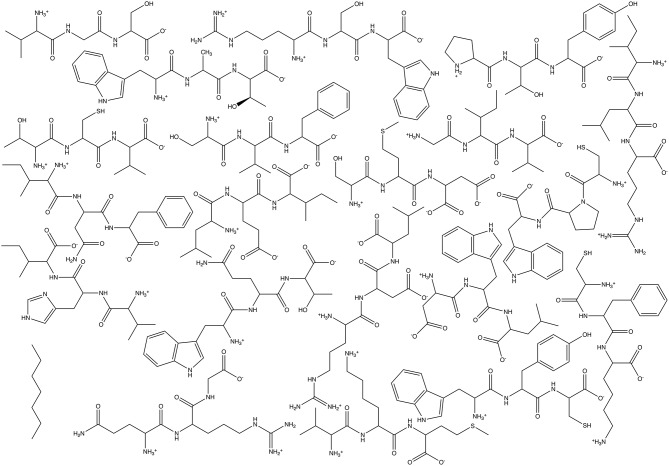
Chemical structures of the molecules used to generate the two Athenaeums used here: 20 tripeptides plus heptane.

For these tests, we utilized simple Athenaeums to better illustrate the effects of Athenaeum content and the choices that a user can make in fragment generation and in running CherryPicker. Two different Athenaeums were generated from a set of 21 small molecules. For each of the 21 natural amino acids, a tripeptide was generated, with charged N- and C- termini and the amino acid flanked by two random amino acids. Generating these 20 random molecules resulted in the following sequences: Val-Gly-Ser, Trp-Ala-Thr, Arg-Ser-Trp, Pro-Thr-Tyr, Thr-Cys-Val, Ser-Val-Phe, Ile-Leu-Arg, Gly-Ile-Val, Ser-Met-Asp, Cys-Pro-Trp, Cys-Phe-Lys, Trp-Tyr-Cys, Asp-Trp-Leu, Leu-Glu-Ile, Ile-Asn-Phe, Trp-Gln-Thr, Val-His-Ile, Val-Lys-Met, and Gln-Arg-Gly. Of note is that the amino acids Asp and Glu were in their deprotonated, negatively charged state, Lys and Arg were in their protonated, positively charge state, and His was neutral and protonated at the NE2 position. The final molecule was heptane. Structures of the molecules used for Athenaeum generation are given in Figure 5. All molecules were in GROMOS united-atom format and parameterized using the standard GROMOS 54A7 force field (Schmid et al., 2011).

**Figure 6:**
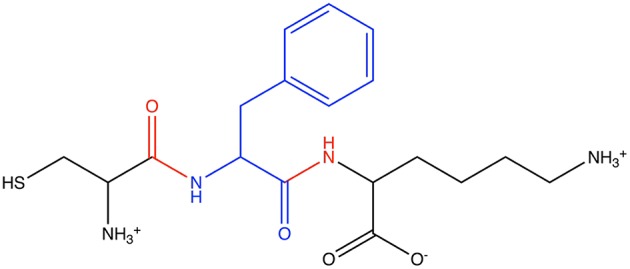
Example of a fragment in the user-specified Athenaeum: the phenylalanine-centered tripeptide Cys-Phe-Lys, showing (blue) the core atoms of the phenylalamine fragment and (red) the overlap regions.

The first Athenaeum was built from a simple set of user-specified fragments. For each of the 20 random peptides, the central amino was defined as the core region of a fragment, and each instance of a terminal amino acid was also defined as the core region of a fragment. In all cases, the overlap region comprised the neighboring amine and/or carboxo group. This resulted in 44 fragments. Figure 6 shows an example of one such fragment from one of the peptides. From heptane, two fragments were defined. The core of the first fragment comprises the three carbon atoms from a methyl terminal, and its overlap region is the next carbon in the chain. The core of the second fragment comprises the three carbon atoms in the center of the heptane molecule, with the overlap region comprising a single carbon atom on either side. This Athenaeum was marked as being self-consistent. While this Athenaeum is sufficient for the test cases presented here, we note that it will not be able to parameterize all possible linear proteins/peptides containing only the natural 20 amino acids as, for example, not all protonation states of side chains are present. However, this is easily resolved by adding more source molecules. On the flip side, a user-specified Athenaeum has the advantage of allowing prior knowledge, such as which functional groups or connections between functional groups are most transferable, to be included.

The second Athenaeum was automatically generated. The 21 molecules listed above were passed through the fully-automated fragment generation method (see above) using an overlap length of *k* = 1, which gave rise to an Athenaeum containing 134799 fragments. It is not marked as being self-consistent, as not all fragments created in this way will provide the same parameters to a given functional group.

### 3.2. Linear Octapeptide

Parameterisation of linear peptides/proteins is already well supported by all molecular dynamics simulation engines. We use this example to illustrate that CherryPicker produces the same results as existing methods, such as the GROMACS (Abraham et al., 2015) tool *pdb2gmx*, which generates parameter files for proteins from a coordinate (PDB) file. Using only the user-specified Athenaeum, CherryPicker exactly reproduces the parameters that *pdb2gmx* provides for the linear octapeptide using the GROMOS 54A7 force field. While it would generally be ill-advised to use CherryPicker in such a manner, as existing tools are more than capable of performing the same task, CherryPicker does have some advantages. For example, *pdb2gmx* uses a database to identify amino acids based on the amino acid names in the input coordinate file. If any of the amino acids are misnamed, *pdb2gmx* is unable to determine which parameters to assign. As CherryPicker uses a chemical structure-matching algorithm, it does not require the input amino acids to be correctly named.

### 3.3. Axinellin A

*Axinellin A* is a cyclic peptide, parameterisation of which is not explicitly supported by *pdb2gmx*. While it is relatively simple to generate parameters for the corresponding linear peptide and then manually modify the resultant parameter file to create a cyclic peptide, this requires detailed understanding of both the parameter file format and the correct parameters to select. CherryPicker, in contrast, handles cyclic and linear systems equivalently.

Using only the user-specified Athenaeum, CherryPicker is unable to completely parameterize *axinellin A*. Specifically, it fails to parameterize the two residues that precede proline residues, that is, the asparagine residue and the penultimate phenylalanine residue. This is not unexpected, as the cyclic nature of the proline side chain means that its backbone amine group is tertiary as opposed to secondary as it is for all the other residues. The simple user-specified Athenaeum does not contain fragments for all the different amino acids preceding a proline, and is thus unable to parameterize all Xxx-Pro combinations.

This failure of the user-specified Athenaeum provides an opportunity to showcase the use of an automatically-generated Athenaeum. From the molecules used to generate the Athenaeums (Figure 5), it is clear that parameters for the two unparameterized residues are available, but the necessary fragments are not included in the user-specified Athenaeum. Adding the automatically-generated Athenaeum, which is searched only after the user-specified Athenaeum and so only used to parameterize regions of the target molecule not parameterized by the user-specified Athenaeum, does result in complete parameterisation of *axinellin A*. The automatically-generated Athenaeum also results in self-consistent parameters in this instance, which are consistent with those generated by manually cyclising a parameter file for a linear version of Axinellin A generated using *pdb2gmx*.

### 3.4. Polymyxin B3

Our final test case is *polymyxin B3*, a peptide that is both cyclic and branched, which makes it difficult to handle with a tool like *pdb2gmx*. Additionally, it contains non-natural amino acids and a lipidated amino acid, parameters for which are not included in the standard force field parameter files provided with molecular dynamics engines and searched by tools like *pdb2gmx*. It is therefore an ideal case for illustration of the functionality of CherryPicker.

For this molecule we ignore the stereochemistry of the C_α_ backbone carbon, i.e., both L- and D-amino acids are considered equivalent. This is easily adjusted by the user after parameter generation, but can also be solved by generating an Athenaeum that includes L- and D-amino acids and using a mask that includes stereochemistry matching.

With the user-directed Athenaeum, CherryPicker is only able to extract parameters for the naturally occurring amino acids, threonine, phenylalanine, and leucine, as expected given the nature of this Athenaeum. It also extracts parameters for the majority of the fatty acid, excluding the peptide bond that joints it to the remainder of the polymyxin. The natural amino acids are correctly identified, and the presence of heptane fragments allows for the identification of straight chain alkane fragments.

After exhausting the fragments in the manual Athenaeum, the remaining unparametrized portions of *polymyxin B3* are mainly the diaminobutyric acid residues. Structurally, these are similar to lysine residues, except with a shorter carbon chain length. As such, an Athenaeum containing fragments of lysine would be expected to be able to parameterize the diaminobutyric acid residues. This is what is observed when using the automatically-generated Athenaeum. All atoms, bonds and angles, except those involving the carboxy group joining the fatty acid tail to the peptide chain, are assigned consistent parameters.

The unmatched atoms, bonds and angles involving the carboxy group of the fatty acid indicate a deficiency of the Athenaeum, rather than a deficiency of the matching algorithm. This can easily be overcome by building additional Athenaeums that incorporate a wider range of already-parameterized molecules, such as lipids. Our purpose here, however, was to illustrate how much can be achieved with even a very simple Athenaeum that requires only molecules already available in standard biomolecular force fields.

Parameters were also not assigned to the dihedral terms for rotation about the central bond of the diaminobutyric acid side chains. This is because the fragments that matched to these side chains had a maximum (core plus overlap) size of three, whereas a core+overlap size of four is required for dihedral terms to be included in a fragment, even utilizing dangling bonds. Again, this would be alleviated by using a broader Athenaeum that covered more chemical groups.

In the current implementation of CherryPicker, placeholder parameters are inserted in the output parameter file when parameters are not assigned after parsing all available Athenaeums, giving the user a clear indication that parameters were not assigned from the Athenaeum. In the future, it will be possible to automatically identify unmatched regions and extract them as separate molecules, capped as necessary to maintain appropriate chemistry, for parameterisation by other means. As these regions are likely to be individually fairly small, parameterisation using automated web servers that carry out quantum chemical calculations will be possible. Once parameterized, these molecules will then be able to be added to the Athenaeum, thus plugging its gaps and broadening the scope of molecules that can be parameterized.

### 3.5. Limitations

As with all automated parameterisation schemes, there are limitations to the approach presented here. Firstly, the quality of parameters obtained for the target molecule is highly dependent on the nature and parameters of the source molecules from which fragments are generated. If molecular dynamics simulations of the source molecules give incorrect emergent properties, simulations of target molecules parameterized by CherryPicker will likely also give incorrect properties. Additionally, deficiencies in the Athenaeum, i.e., Athenaeums that do not provide fragments to cover all regions of a target molecule, will become apparent, as was shown with the parameterisation of *polymyxin B3* discussed above. Finally, the choice of overlap size will have a potentially pronounced effect on the parameters attained, especially in the fully automated use case. A smaller overlap will lead to more of a target molecule being parameterized, but risks loss of the chemical environment selectivity that a larger overlap region enables, and thus poorer quality parameters.

## 4. Conclusions

The CherryPicker algorithm, combined with one or more Athenaeums, provides a simple to use yet widely applicable method for rapidly generating parameters for novel biomolecules. By assembling parameters derived from fragments of molecules that are already parameterized according to a particular biomolecular force field, we ensure that the resultant parameter set for the novel molecule is by design compatible with an existing force field. The user is able to specify the nature and number of Athenaeums used, including how the already-parameterized molecules are fragmented. This provides control over, for instance, the reliability and consistency of the parameters that are assigned to the target molecule. In demonstrating the application of CherryPicker to a series of peptides of varying degrees of complexity, we illustrate how even very simple Athenaeums are able to parameterize a wider range of molecules than would be possible with existing parameter generation tools. Target molecules are input to CherryPicker in the standard and commonly-used PDB format, and coordinate and parameter files are output in the formats required for the popular GROMACS simulation software, making it straightforward to integrate CherryPicker into established simulation pipelines. This will in turn act to facilitate the use of molecular simulations across a broad range of scientific fields.

## Author Contributions

IW co-designed, coded, and tested the software and co-wrote the manuscript. JA co-designed the software and tests, and co-wrote the paper.

### Conflict of Interest Statement

The authors declare that the research was conducted in the absence of any commercial or financial relationships that could be construed as a potential conflict of interest.

## References

[B1] Abraham M. J. Murtola T. Schulz R. Páll S. Smith J. C. Hess B. (2015). Gromacs: high performance molecular simulations through multi-level parallelism from laptops to supercomputers. SoftwareX 1–2:19–25. 10.1016/j.softx.2015.06.001

[B2] Betz R. M. Walker R. C. (2015). Paramfit: automated optimization of force field parameters for molecular dynamics simulations. J. Comput. Chem. 36, 79–87. 10.1002/jcc.23775 25413259

[B3] Bonnici V. Giugno R. Pulvirenti A. Shasha D. Ferro A. (2013). A subgraph isomorphism algorithm and its application to biochemical data. BMC Bioinformat. 14:S13. 10.1186/1471-2105-14-S7-S13 23815292 PMC3633016

[B4] Brooks B. R. Bruccoleri R. E. Olafson B. D. States D. J. Swaminathan S. Karplus M. (1983). Charmm: a program for macromolecular energy, minimization, and dynamics calculations. J. Comput. Chem. 4, 187–217.

[B5] Canzar S. El-Kebir M. Pool R. Elbassioni K. Malde A. K. Mark A. E.. (2013). Charge group partitioning in biomolecular simulation. J. Comput. Biol. 20, 188–198. 10.1089/cmb.2012.0239 23461571 PMC3590896

[B6] Christen M. Hünenberger P. H. Bakowies D. Baron R. Bürgi R. Geerke D. P.. (2005). The gromos software for biomolecular simulation: Gromos05. J. Comput. Chem. 26, 1719–1751. 10.1002/jcc.20303 16211540

[B7] Cordella L. P. Foggia P. Sansone C. Vento M. (2004). A (sub)graph isomorphism algorithm for matching large graphs. IEEE Trans. Pattern Anal. Mach. Intell. 26, 1367–1372. 10.1109/TPAMI.2004.75 15641723

[B8] Cornell W. D. Cieplak P. Bayly C. I. Gould I. R. Merz K. M. Ferguson D. M. (1995). A second generation force field for the simulation of proteins, nucleic acids, and organic molecules. J. Am. Chem. Soc. 117, 5179–5197.

[B9] Dodda L. S. Cabeza de Vaca I. Tirado-Rives J. Jorgensen W. L. (2017). Ligpargen web server: an automatic opls-aa parameter generator for organic ligands. Nucleic Acids Res. 45, W331–W336. 10.1093/nar/gkx312 28444340 PMC5793816

[B10] Duan Y. Wu C. Chowdhury S. Lee M. C. Xiong G. Zhang W.. (2003). A point-charge force field for molecular mechanics simulations of proteins based on condensed-phase quantum mechanical calculations. J. Comput. Chem. 24, 1999–2012. 14531054 10.1002/jcc.10349

[B11] Fairweather K. A. Sayyadi N. Roussakis C. Jolliffe K. A. (2010). Synthesis of the cyclic heptapeptide axinellin a. Tetrahedron 66, 935–939. 10.1016/j.tet.2009.11.090

[B12] Foloppe N. MacKerell A. D. Jr (2000). All-atom empirical force field for nucleic acids: I. parameter optimization based on small molecule and condensed phase macromolecular target data. J. Comput. Chem. 21, 86–104.

[B13] Jakob W. Rhinelander J. Moldovan D. (2017). Pybind11–Seamless Operability Between c++11 and Python. Available online at: https://github.com/pybind/pybind11 (accessed September 11, 2018).

[B14] Kaminski G. A. Friesner R. A. Tirado-Rives J. Jorgensen W. L. (2001). Evaluation and reparametrization of the opls-aa force field for proteins via comparison with accurate quantum chemical calculations on peptides. J. Phys. Chem. B 105, 6474–6487. 10.1021/jp003919d

[B15] Koch U. Stone A. J. (1996). Conformational dependence of the molecular charge distribution and its influence on intermolecular interactions. J. Chem. Soc. Faraday Trans. 92, 1701–1708. 10.1039/ft9969201701

[B16] Koziara K. Stroet M. Malde A. Mark A. (2014). Testing and validation of the automated topology builder (atb) version 2.0: prediction of hydration free enthalpies. J. Comput. Aided Mol. Design 28, 221–233. 10.1007/s10822-014-9713-7 24477799

[B17] MacKerell A. D. Banavali N. Foloppe N. (2000). Development and current status of the charmm force field for nucleic acids. Biopolymers 56, 257–265. 10.1002/1097-0282(2000)56:4<257::AID-BIP10029>3.0.CO;2-W 11754339

[B18] MacKerell A. D. Bashford D. Bellott M. Dunbrack R. L. Evanseck J. D. Field M. J.. (1998). All-atom empirical potential for molecular modeling and dynamics studies of proteins. J. Phys. Chem. B 102, 3586–3616. 24889800 10.1021/jp973084f

[B19] Malde A. K. Zuo L. Breeze M. Stroet M. Poger D. Nair P. C.. (2011). An automated force field topology builder (atb) and repository: Version 1.0. J. Chem. Theory Comput. 7, 4026–4037. 10.1021/ct200196m 26598349

[B20] Miller B. T. Singh R. P. Klauda J. B. Hodošček M. Brooks B. R. Woodcock H. L. (2008). Charmming: a new, flexible web portal for charmm. J. Chem. Inform. Model. 48, 1920–1929. 10.1021/ci800133b 18698840 PMC2676146

[B21] Oostenbrink C. Villa A. Mark A. E. Van Gunsteren W. F. (2004). A biomolecular force field based on the free enthalpy of hydration and solvation: the gromos force-field parameter sets 53a5 and 53a6. J. Comput. Chem. 25, 1656–1676. 10.1002/jcc.20090 15264259

[B22] Poger D. Van Gunsteren W. F. Mark A. E. (2010). A new force field for simulating phosphatidylcholine bilayers. J. Comput. Chem. 31, 1117–1125. 10.1002/jcc.21396 19827145

[B23] Randazzo A. Dal Piaz F. Orrù S. Debitus C. Roussakis C. Pucci P. (1998). Axinellins a and b: New proline-containing antiproliferative cyclopeptides from the vanuatu sponge axinella carteri. Eur. J. Organ. Chem. 1998, 2659–2665.

[B24] Reiher W. E. (1985). Theoretical Studies of Hydrogen Bonding. Ph.D. thesis, Harvard University. “Theoretical Studies of Hydrogen Bonding.”

[B25] Ribeiro A. A. S. T. Horta B. A. C. de Alencastro R. B. (2008). Mktop: a program for automatic construction of molecular topologies. J. Brazil. Chem. Soc. 19, 1433–1435. 10.1590/S0103-50532008000700031

[B26] Schmid N. Eichenberger A. Choutko A. Riniker S. Winger M. Mark A.. (2011). Definition and testing of the gromos force-field versions 54a7 and 54b7. Eur. Biophys. J. 40, 843–856. 10.1007/s00249-011-0700-9 21533652

[B27] Schuler L. D. Daura X. van Gunsteren W. F. (2001). An improved gromos96 force field for aliphatic hydrocarbons in the condensed phase. J. Comput. Chem. 22, 1205–1218. 10.1002/jcc.1078.abs

[B28] Schüttelkopf A. W. van Aalten D. M. F. (2004). Prodrg: a tool for high-throughput crystallography of protein–ligand complexes. Acta Crystallogra. Sect. D 60, 1355–1363. 10.1107/S0907444904011679 15272157

[B29] Siek J. Lee L.-Q. Lumsdaine A. (2002). The Boost Graph Library: User Guide and Reference Manual. Boston, MA: Addison-Wesley Longman Publishing Co., Inc.

[B30] Sterling T. Irwin J. J. (2015). Zinc 15–ligand discovery for everyone. J. Chem. Inform. Model. 55, 2324–2337. 10.1021/acs.jcim.5b00559 26479676 PMC4658288

[B31] Stouch T. Williams D. E. (1992). Conformational dependence of electrostatic potential derived charges of a lipid headgroup: Glycerylphosphorylcholine. J. Comput. Chem. 13, 622–632.

[B32] Stouch T. R. Williams D. E. (1993). Conformational dependence of electrostatic potential-derived charges: Studies of the fitting procedure. J. Comput. Chem. 14, 858–866.

[B33] Urban J. J. Famini G. R. (1993). Conformational dependence of the electrostatic potential-derived charges of dopamine: ramifications in molecular mechanics force field calculations in the gas phase and in aqueous solution. J. Comput. Chem. 14, 353–362.

[B34] Vanquelef E. Simon S. Marquant G. Garcia E. Klimerak G. Delepine J. C.. (2011). R.e.d. server: a web service for deriving resp and esp charges and building force field libraries for new molecules and molecular fragments. Nucleic Acids Res. 39(Suppl-2):W511–W517. 10.1093/nar/gkr288 21609950 PMC3125739

[B35] Wang J. Wang W. Kollman P. A. Case D. A. (2006). Automatic atom type and bond type perception in molecular mechanical calculations. J. Mol. Grap. Model. 25, 247–260. 10.1016/j.jmgm.2005.12.005 16458552

[B36] Wang J. Wolf R. M. Caldwell J. W. Kollman P. A. Case D. A. (2004). Development and testing of a general amber force field. J. Comput. Chem. 25, 1157–1174. 10.1002/jcc.20035 15116359

[B37] Weiner S. J. Kollman P. A. Case D. A. Singh U. C. Ghio C. Alagona G. Profeta S. (1984). A new force field for molecular mechanical simulation of nucleic acids and proteins. J. Am. Chem. Soc. 106, 765–784.

[B38] Weiner S. J. Kollman P. A. Nguyen D. T. Case D. A. (1986). An all atom force field for simulations of proteins and nucleic acids. J. Comput. Chem. 7, 230–252. 29160584 10.1002/jcc.540070216

[B39] Welsh I. D. Allison J. R. (2019). Automated simultaneous assignment of bond orders and formal charges. J. Cheminformat. 11:18. 10.1186/s13321-019-0340-0 30840171 PMC6419789

[B40] Zavascki A. Goldani L. Li J. Nation R. (2007). Polymyxin b for the treatment of multidrug-resistant pathogens: a critical review. J. Antimicrob. Chemother. 60, 1206–1215. 10.1093/jac/dkm357 17878146

