# Supplementary material for: CherryPicker: An Algorithm for the Automated Parametrization of Large Biomolecules for Molecular Simulation
Source: Front Chem. 2019 Jun 5;7:400. doi: 10.3389/fchem.2019.00400 (PMC6560068; doi:10.3389/fchem.2019.00400)
Supplement: Supplementary file 1 [file Data_Sheet_1.PDF]

Supplementary Material: CherryPicker: An Algorithm for the Automated Parameterisation of Large Biomolecules for Molecular Simulation

1 CHOICE OF OVERLAP SIZE

The size of the *overlap region* included in a fragment is a property of the Athenaeum to which that fragment belongs, and is chosen when generating fragments. It plays a major role in the ability of an Athenaeum to map fragments to a target molecule, and the reliability of the resulting parameters. The effect of overlap size on the quality of parameters produced by the CherryPicker algorithm was investigated for both the bonded and non-bonded terms.

To investigate the effect on the bonded terms, four Athenaeums with overlap sizes from zero to three atoms inclusive were generated from a collection of 9000 previously parameterised molecules. Each Athenaeum was then mapped to 33 large biomolecules with a lower fragment size limit of five atoms. The number of mapped bond parameter types (where a bond parameter type refers to a particular combination of bond length and force constant) for each bond in the 33 biomolecules were counted (figure S1). With an overlap size of zero, the distribution of the number of matched parameter types is very broad, with a high mean value of 13.2. With an overlap of one, the disitribution becomes concentrated at lower numbers of matched parameter types, but still has a long tail. Overlaps of two and three atoms continue this trend, with increased concentration of the distribution at lower numbers of matched parameter types, and a reduced tail. Generally speaking, a large number of mapped bonded parameter types is undesirable as such a situation indicates that fragments are being mapped that poorly match the surrounding environment. This is most evident with an overlap size of zero, where the environment around a fragment is ignored in the mapping process. The long tail of the distributions with overlap one show that even though having an overlap of one is a vast improvement on having no overlap, a larger overlap of size two or three is preferable.

To investigate the effect of overlap size on the non-bonded terms, namely the atomic point charges, a slightly different approach was taken. Instead of generating an Athenaeum and performing mapping to a target molecule, three fragments with a core size of one atom and with overlap sizes of zero, one, and two atoms were chosen and mapped to all of the 9000 molecules used to generate the Athenaeums described above. While a single atom is not a good choice for the core size, this approach provides a simple means to investigate the effect of increasing overlap size. These mappings were performed considering only the element and formal charge for vertices, and the bond order for edges. The single-atom fragment cores were: the hydrogen atom of a methyl group bonded to another carbon atom; a carbon atom within an aromatic carbon ring, with a hydrogen atom substituent; and the neutral oxyge atomn of a negatively charged carboxylic acid functional group, bonded to another carbon atom. For each combination of core atom type and overlap size, the distributions of point charges for all mapped single atoms across the 9000 molecules are shown in figure S2.

With an overlap size of zero we see that the distribution of point charges for the methyl hydrogen atom is bimodal (figure S2a) due to the inability of a fragment with zero overlap to distinguish between polar and non-polar hydrogen atoms. The distribution of partial atomic charges for the aromatic carbon atom

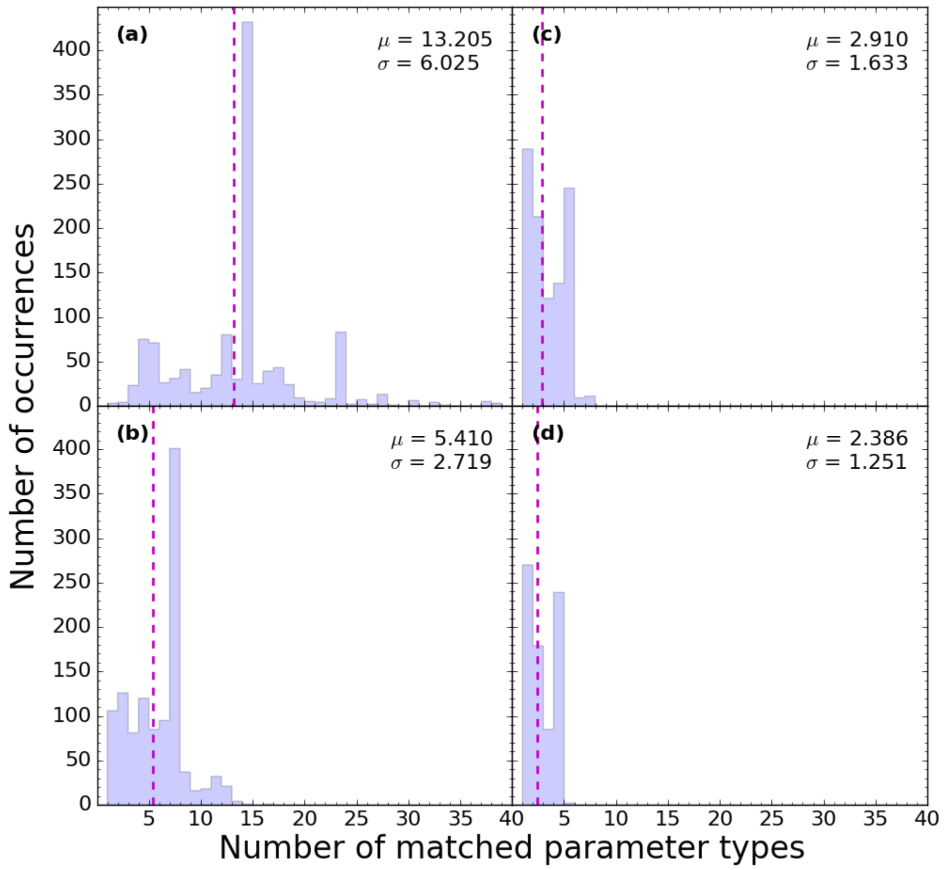

Figure S1. Distributions of the number of bond parameter types mapped to a collection of 33 large molecules using Athenaeums with overlap sizes between (a) 0 and (d) 3. The vertical magenta dashed line shows the mean number of matched bond parameter types for each Athenaeum. Numerical values for the mean (μ) and standard deviation (σ) of each distribution are provided.

is skewed towards negative values (figure S2d), and the distribution of partial atomic charges for the carboxylic oxygen atom has a broad base with a sharp central peak (figure S2g). All distributions have a reasonably large difference between their mean and median values. With an overlap size of one, the distributions are narrower and mean and median values are more similar. The methyl hydrogen atom partial charge distribution becomes unimodal as polar and non-polar hydrogen atoms are now able to be distinguished, however the carboxylic oxygen atom distribution becomes bimodal, due to being unable to distinguish between oxygen atoms in protonated carboxylic acids and deprotonated carboxylic acids. Finally, with an overlap size of two, all distributions are monomodal, narrow and shapr with a relatively small standard deviation and almost identical mean and median values.

Together, these investigations into the effect of overlap size on the resulting bonded and non-bonded parameters indicate that an overlap size of at least two is most appropriate. It should be noted, however, that the larger the overlap size, the fewer fragments will map to a given target molecule. Thus while the safest approach is to use a larger overlap size of three or more atoms, in some cases, it may be necessary to reduce this to obtain parameter estimates. Such parameters should be treated with care, however, and tested thoroughly before use.

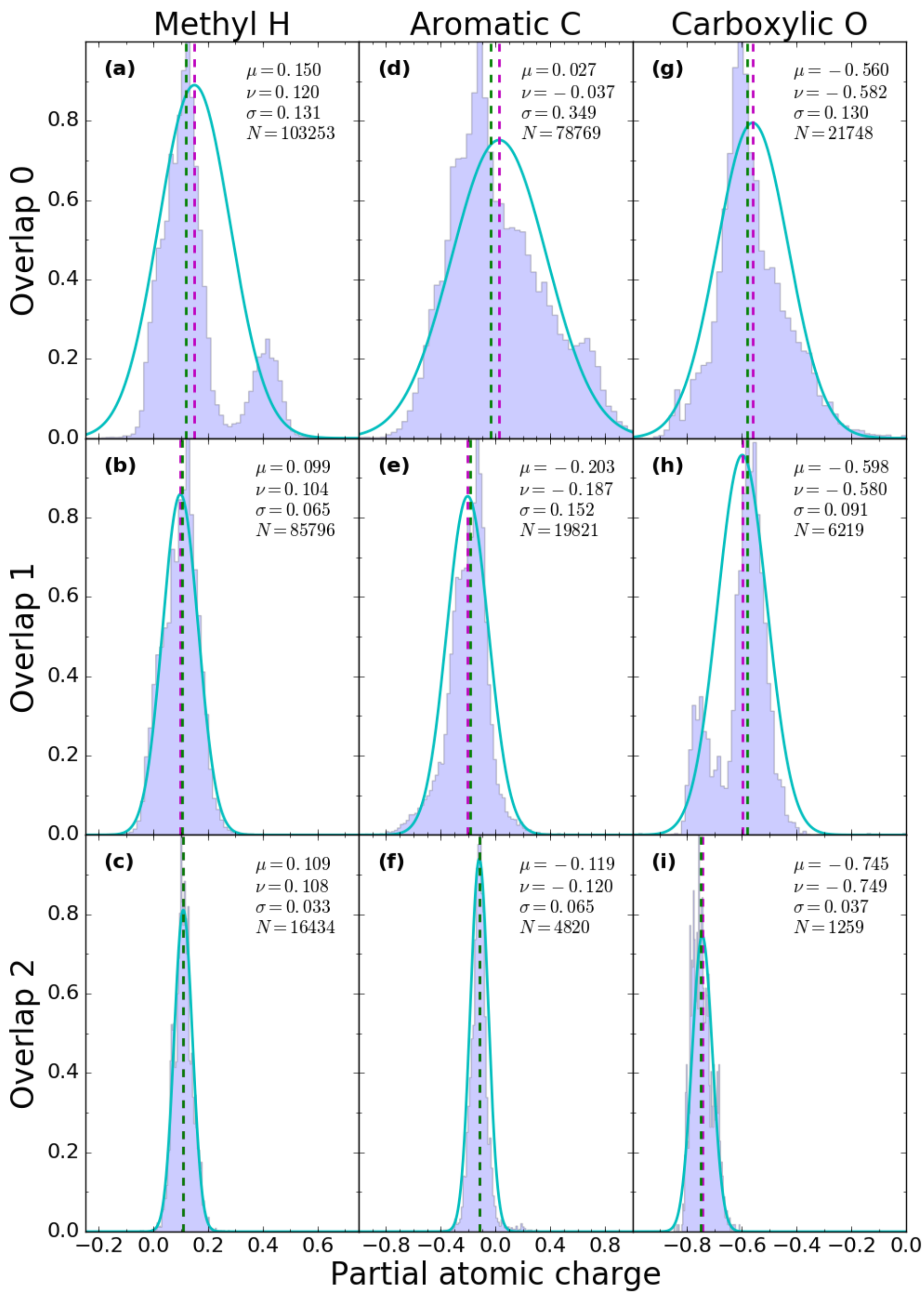

Figure S2. Distributions of partial atomic charges mapped to target fragments of core size one and with varying overlap sizes as labelled. The central atom of each fragment was (a) to (c) a methyl hydrogen atom, (d) to (f) an aromatic carbon atom, and (g) to (i) a carboxylic acid oxygen atom. The cyan curves are the Gaussian function using the mean (μ) and standard deviation (σ) of the underlying histogram. Dashed vertical lines represent (magenta) μ and (green) the median (ν) of the distribution. The counts (y-axis) have been normalised so that the largest count is 1.0 for ease of comparison, with the actual counts (N) given.

2 MAPPED PARAMETERS

2.1 Linear octapeptide

Parameters obtained using only the manually specified Athenaeum. Everything has been correctly mapped.

```
[ atoms ]
 1     NL     1     TEST       N     1     0.12900   14.0067
 2      H     1     TEST       H     2     0.24800    1.0080
 3      H     1     TEST       H     3     0.24800    1.0080
 4      H     1     TEST       H     4     0.24800    1.0080
 5    CH1     1     TEST       C     5     0.12700   13.0190
 6    CH2     1     TEST       C     6     0.00000   14.0269
 7      C     1     TEST       C     7     0.45000   12.0110
 8    CH2     1     TEST       C     8     0.00000   14.0269
 9      O     1     TEST       O     9    -0.45000   15.9994
10    CH2     1     TEST       C    10     0.09000   14.0269
11     NE     1     TEST       N    11    -0.11000   14.0067
12      H     1     TEST       H    12     0.24000    1.0080
13      C     1     TEST       C    13     0.34000   12.0110
14     NZ     1     TEST       N    14    -0.26000   14.0067
15     NZ     1     TEST       N    15    -0.26000   14.0067
16      H     1     TEST       H    16     0.24000    1.0080
17      H     1     TEST       H    17     0.24000    1.0080
18      H     1     TEST       H    18     0.24000    1.0080
19      H     1     TEST       H    19     0.24000    1.0080
20      N     1     TEST       N    20    -0.31000   14.0067
21      H     1     TEST       H    21     0.31000    1.0080
22    CH2     1     TEST       C    22     0.00000   14.0269
23      C     1     TEST       C    23     0.45000   12.0110
24      O     1     TEST       O    24    -0.45000   15.9994
25      N     1     TEST       N    25    -0.31000   14.0067
26      H     1     TEST       H    26     0.31000    1.0080
27    CH1     1     TEST       C    27     0.00000   13.0190
28    CH2     1     TEST       C    28     0.26600   14.0269
29      C     1     TEST       C    29     0.45000   12.0110
30     OA     1     TEST       O    30    -0.67400   15.9994
31      O     1     TEST       O    31    -0.45000   15.9994
32      H     1     TEST       H    32     0.40800    1.0080
33      N     1     TEST       N    33    -0.31000   14.0067
34      H     1     TEST       H    34     0.31000    1.0080
35    CH1     1     TEST       C    35     0.00000   13.0190
36    CH1     1     TEST       C    36     0.00000   13.0190
37      C     1     TEST       C    37     0.45000   12.0110
38    CH3     1     TEST       C    38     0.00000   15.0349
39    CH3     1     TEST       C    39     0.00000   15.0349
40      O     1     TEST       O    40    -0.45000   15.9994
41      N     1     TEST       N    41    -0.31000   14.0067
42      H     1     TEST       H    42     0.31000    1.0080
43    CH1     1     TEST       C    43     0.00000   13.0190
44    CH2     1     TEST       C    44     0.00000   14.0269
```

45	C	1	TEST	C	45	0.45000	12.0110
46	CH2	1	TEST	C	46	0.00000	14.0269
47	O	1	TEST	O	47	-0.45000	15.9994
48	CH2	1	TEST	C	48	0.00000	14.0269
49	CH2	1	TEST	C	49	0.12700	14.0269
50	NL	1	TEST	N	50	0.12900	14.0067
51	H	1	TEST	H	51	0.24800	1.0080
52	H	1	TEST	H	52	0.24800	1.0080
53	H	1	TEST	H	53	0.24800	1.0080
54	N	1	TEST	N	54	-0.31000	14.0067
55	H	1	TEST	H	55	0.31000	1.0080
56	CH1	1	TEST	C	56	0.00000	13.0190
57	CH2	1	TEST	C	57	0.26600	14.0269
58	C	1	TEST	C	58	0.45000	12.0110
59	OA	1	TEST	O	59	-0.67400	15.9994
60	O	1	TEST	O	60	-0.45000	15.9994
61	H	1	TEST	H	61	0.40800	1.0080
62	N	1	TEST	N	62	-0.31000	14.0067
63	H	1	TEST	H	63	0.31000	1.0080
64	CH1	1	TEST	C	64	0.00000	13.0190
65	CH2	1	TEST	C	65	0.00000	14.0269
66	C	1	TEST	C	66	0.45000	12.0110
67	C	1	TEST	C	67	-0.21000	12.0110
68	O	1	TEST	O	68	-0.45000	15.9994
69	C	1	TEST	C	69	-0.14000	12.0110
70	C	1	TEST	C	70	0.00000	12.0110
71	HC	1	TEST	H	71	0.14000	1.0080
72	NR	1	TEST	N	72	-0.10000	14.0067
73	C	1	TEST	C	73	0.00000	12.0110
74	C	1	TEST	C	74	-0.14000	12.0110
75	H	1	TEST	H	75	0.31000	1.0080
76	C	1	TEST	C	76	-0.14000	12.0110
77	C	1	TEST	C	77	-0.14000	12.0110
78	HC	1	TEST	H	78	0.14000	1.0080
79	HC	1	TEST	H	79	0.14000	1.0080
80	C	1	TEST	C	80	-0.14000	12.0110
81	HC	1	TEST	H	81	0.14000	1.0080
82	HC	1	TEST	H	82	0.14000	1.0080
83	N	1	TEST	N	83	-0.31000	14.0067
84	H	1	TEST	H	84	0.31000	1.0080
85	CH1	1	TEST	C	85	0.00000	13.0190
86	CH2	1	TEST	C	86	0.00000	14.0269
87	C	1	TEST	C	87	0.27000	12.0110
88	C	1	TEST	C	88	0.00000	12.0110
89	OM	1	TEST	O	89	-0.63500	15.9994

```
90      OM      1      TEST        O     90     -0.63500   15.9994
91       C      1      TEST        C     91     -0.14000   12.0110
92       C      1      TEST        C     92     -0.14000   12.0110
93      HC      1      TEST        H     93      0.14000    1.0080
94       C      1      TEST        C     94     -0.14000   12.0110
95       C      1      TEST        C     95     -0.14000   12.0110
96      HC      1      TEST        H     96      0.14000    1.0080
97      HC      1      TEST        H     97      0.14000    1.0080
98       C      1      TEST        C     98     -0.14000   12.0110
99      HC      1      TEST        H     99      0.14000    1.0080
100      HC      1      TEST        H    100      0.14000    1.0080

[ bonds ]
 1      2      2     gb_2
 1      3      2     gb_2
 1      4      2     gb_2
 1      5      2     gb_21
 5      6      2     gb_27
 5      7      2     gb_27
 6      8      2     gb_27
 7      9      2     gb_5
 7     20      2     gb_10
 8     10      2     gb_27
10     11      2     gb_21
11     12      2     gb_2
11     13      2     gb_11
13     14      2     gb_11
13     15      2     gb_11
14     16      2     gb_2
14     17      2     gb_2
15     18      2     gb_2
15     19      2     gb_2
20     21      2     gb_2
20     22      2     gb_21
22     23      2     gb_27
23     24      2     gb_5
23     25      2     gb_10
25     26      2     gb_2
25     27      2     gb_21
27     28      2     gb_27
27     29      2     gb_27
28     30      2     gb_18
29     31      2     gb_5
29     33      2     gb_10
30     32      2     gb_1
```

33	34	2	gb_2
33	35	2	gb_21
35	36	2	gb_27
35	37	2	gb_27
36	38	2	gb_27
36	39	2	gb_27
37	40	2	gb_5
37	41	2	gb_10
41	42	2	gb_2
41	43	2	gb_21
43	44	2	gb_27
43	45	2	gb_27
44	46	2	gb_27
45	47	2	gb_5
45	54	2	gb_10
46	48	2	gb_27
48	49	2	gb_27
49	50	2	gb_21
50	51	2	gb_2
50	52	2	gb_2
50	53	2	gb_2
54	55	2	gb_2
54	56	2	gb_21
56	57	2	gb_27
56	58	2	gb_27
57	59	2	gb_18
58	60	2	gb_5
58	62	2	gb_10
59	61	2	gb_1
62	63	2	gb_2
62	64	2	gb_21
64	65	2	gb_27
64	66	2	gb_27
65	67	2	gb_27
66	68	2	gb_5
66	83	2	gb_10
67	69	2	gb_10
67	70	2	gb_16
69	71	2	gb_3
69	72	2	gb_10
70	73	2	gb_16
70	74	2	gb_16
72	73	2	gb_10
72	75	2	gb_2
73	76	2	gb_16

```
74    77      2    gb_16
74    78      2    gb_3
76    79      2    gb_3
76    80      2    gb_16
77    80      2    gb_16
77    81      2    gb_3
80    82      2    gb_3
83    84      2    gb_2
83    85      2    gb_21
85    86      2    gb_27
85    87      2    gb_27
86    88      2    gb_27
87    89      2    gb_6
87    90      2    gb_6
88    91      2    gb_16
88    92      2    gb_16
91    93      2    gb_3
91    94      2    gb_16
92    95      2    gb_16
92    96      2    gb_3
94    97      2    gb_3
94    98      2    gb_16
95    98      2    gb_16
95    99      2    gb_3
98   100      2    gb_3

[ angles ]
 2     1     3     2    ga_10
 2     1     4     2    ga_10
 2     1     5     2    ga_11
 3     1     4     2    ga_10
 3     1     5     2    ga_11
 4     1     5     2    ga_11
 1     5     6     2    ga_13
 1     5     7     2    ga_13
 6     5     7     2    ga_13
 5     6     8     2    ga_15
 5     7     9     2    ga_30
 5     7    20     2    ga_19
 9     7    20     2    ga_33
 6     8    10     2    ga_15
 8    10    11     2    ga_13
10    11    12     2    ga_20
10    11    13     2    ga_33
12    11    13     2    ga_23
```

11	13	14	2	ga_28
11	13	15	2	ga_28
14	13	15	2	ga_28
13	14	16	2	ga_23
13	14	17	2	ga_23
16	14	17	2	ga_24
13	15	18	2	ga_23
13	15	19	2	ga_23
18	15	19	2	ga_24
7	20	21	2	ga_32
7	20	22	2	ga_31
21	20	22	2	ga_18
20	22	23	2	ga_13
22	23	24	2	ga_30
22	23	25	2	ga_19
24	23	25	2	ga_33
23	25	26	2	ga_32
23	25	27	2	ga_31
26	25	27	2	ga_18
25	27	28	2	ga_13
25	27	29	2	ga_13
28	27	29	2	ga_13
27	28	30	2	ga_13
27	29	31	2	ga_30
27	29	33	2	ga_19
31	29	33	2	ga_33
28	30	32	2	ga_12
29	33	34	2	ga_32
29	33	35	2	ga_31
34	33	35	2	ga_18
33	35	36	2	ga_13
33	35	37	2	ga_13
36	35	37	2	ga_13
35	36	38	2	ga_15
35	36	39	2	ga_15
38	36	39	2	ga_15
35	37	40	2	ga_30
35	37	41	2	ga_19
40	37	41	2	ga_33
37	41	42	2	ga_32
37	41	43	2	ga_31
42	41	43	2	ga_18
41	43	44	2	ga_13
41	43	45	2	ga_13
44	43	45	2	ga_13

43	44	46	2	ga_15
43	45	47	2	ga_30
43	45	54	2	ga_19
47	45	54	2	ga_33
44	46	48	2	ga_15
46	48	49	2	ga_15
48	49	50	2	ga_15
49	50	51	2	ga_11
49	50	52	2	ga_11
49	50	53	2	ga_11
51	50	52	2	ga_10
51	50	53	2	ga_10
52	50	53	2	ga_10
45	54	55	2	ga_32
45	54	56	2	ga_31
55	54	56	2	ga_18
54	56	57	2	ga_13
54	56	58	2	ga_13
57	56	58	2	ga_13
56	57	59	2	ga_13
56	58	60	2	ga_30
56	58	62	2	ga_19
60	58	62	2	ga_33
57	59	61	2	ga_12
58	62	63	2	ga_32
58	62	64	2	ga_31
63	62	64	2	ga_18
62	64	65	2	ga_13
62	64	66	2	ga_13
65	64	66	2	ga_13
64	65	67	2	ga_15
64	66	68	2	ga_30
64	66	83	2	ga_19
68	66	83	2	ga_33
65	67	69	2	ga_37
65	67	70	2	ga_37
69	67	70	2	ga_7
67	69	71	2	ga_36
67	69	72	2	ga_7
71	69	72	2	ga_36
67	70	73	2	ga_7
67	70	74	2	ga_39
73	70	74	2	ga_27
69	72	73	2	ga_7
69	72	75	2	ga_36

73	72	75	2	ga_36
70	73	72	2	ga_7
70	73	76	2	ga_27
72	73	76	2	ga_39
70	74	77	2	ga_27
70	74	78	2	ga_25
77	74	78	2	ga_25
73	76	79	2	ga_25
73	76	80	2	ga_27
79	76	80	2	ga_25
74	77	80	2	ga_27
74	77	81	2	ga_25
80	77	81	2	ga_25
76	80	77	2	ga_27
76	80	82	2	ga_25
77	80	82	2	ga_25
66	83	84	2	ga_32
66	83	85	2	ga_31
84	83	85	2	ga_18
83	85	86	2	ga_13
83	85	87	2	ga_13
86	85	87	2	ga_13
85	86	88	2	ga_15
85	87	89	2	ga_22
85	87	90	2	ga_22
89	87	90	2	ga_38
86	88	91	2	ga_27
86	88	92	2	ga_27
91	88	92	2	ga_27
88	91	93	2	ga_25
88	91	94	2	ga_27
93	91	94	2	ga_25
88	92	95	2	ga_27
88	92	96	2	ga_25
95	92	96	2	ga_25
91	94	97	2	ga_25
91	94	98	2	ga_27
97	94	98	2	ga_25
92	95	98	2	ga_27
92	95	99	2	ga_25
98	95	99	2	ga_25
94	98	95	2	ga_27
94	98	100	2	ga_25
95	98	100	2	ga_25

```
[ dihedrals ]
 2     1     5     7     1     gd_39
 1     5     6     8     1     gd_34
 1     5     7    20     1     gd_42
 1     5     7    20     1     gd_45
 5     6     8    10     1     gd_34
 5     7    20    22     1     gd_14
 6     8    10    11     1     gd_34
 8    10    11    13     1     gd_39
10    11    13    14     1     gd_14
11    13    14    16     1     gd_14
11    13    15    18     1     gd_14
 7    20    22    23     1     gd_43
 7    20    22    23     1     gd_44
20    22    23    25     1     gd_42
20    22    23    25     1     gd_45
22    23    25    27     1     gd_14
23    25    27    29     1     gd_43
23    25    27    29     1     gd_44
25    27    28    30     1     gd_34
25    27    29    33     1     gd_42
25    27    29    33     1     gd_45
27    28    30    32     1     gd_23
27    29    33    35     1     gd_14
29    33    35    37     1     gd_43
29    33    35    37     1     gd_44
33    35    36    38     1     gd_34
33    35    36    39     1     gd_34
33    35    37    41     1     gd_42
33    35    37    41     1     gd_45
35    37    41    43     1     gd_14
37    41    43    45     1     gd_43
37    41    43    45     1     gd_44
41    43    44    46     1     gd_34
41    43    45    54     1     gd_42
41    43    45    54     1     gd_45
43    44    46    48     1     gd_34
43    45    54    56     1     gd_14
44    46    48    49     1     gd_34
46    48    49    50     1     gd_34
48    49    50    51     1     gd_29
45    54    56    58     1     gd_43
45    54    56    58     1     gd_44
54    56    57    59     1     gd_34
54    56    58    62     1     gd_42
```

54	56	58	62	1	gd_45
56	57	59	61	1	gd_23
56	58	62	64	1	gd_14
58	62	64	66	1	gd_43
58	62	64	66	1	gd_44
62	64	65	67	1	gd_34
62	64	66	83	1	gd_42
62	64	66	83	1	gd_45
64	65	67	70	1	gd_40
64	66	83	85	1	gd_14
70	67	69	72	2	gi_1
69	67	70	73	2	gi_1
67	69	72	73	2	gi_1
67	70	73	72	2	gi_1
74	70	73	76	2	gi_1
73	70	74	77	2	gi_1
69	72	73	70	2	gi_1
70	73	76	80	2	gi_1
70	74	77	80	2	gi_1
73	76	80	77	2	gi_1
74	77	80	76	2	gi_1
66	83	85	87	1	gd_43
66	83	85	87	1	gd_44
83	85	86	88	1	gd_34
83	85	87	90	1	gd_42
83	85	87	90	1	gd_45
85	86	88	91	1	gd_40
85	86	88	92	1	gd_40
92	88	91	94	2	gi_1
91	88	92	95	2	gi_1
88	91	94	98	2	gi_1
88	92	95	98	2	gi_1
91	94	98	95	2	gi_1
92	95	98	94	2	gi_1
37	35	41	40	2	gi_1
35	38	39	36	2	gi_2
35	33	37	36	2	gi_2
34	35	29	33	2	gi_1
35	39	38	36	2	gi_2
87	85	90	89	2	gi_1
98	94	95	100	2	gi_1
95	98	92	99	2	gi_1
94	98	91	97	2	gi_1
92	88	95	96	2	gi_1
91	88	94	93	2	gi_1

```
86    92    91    88     2    gi_1
85    83    87    86     2    gi_2
83    66    85    84     2    gi_1
98    95    94   100     2    gi_1
86    91    92    88     2    gi_1
20     7    22    21     2    gi_1
 7     5    20     9     2    gi_1
23    22    25    24     2    gi_1
13    18    19    15     2    gi_1
13    17    16    14     2    gi_1
11    15    14    13     2    gi_1
11    10    13    12     2    gi_1
 5     1     7     6     2    gi_2
13    16    17    14     2    gi_1
13    19    18    15     2    gi_1
29    27    33    31     2    gi_1
27    25    29    28     2    gi_2
25    23    27    26     2    gi_1
58    56    62    60     2    gi_1
56    54    58    57     2    gi_2
54    45    56    55     2    gi_1
45    43    54    47     2    gi_1
43    41    45    44     2    gi_2
41    37    43    42     2    gi_1
66    64    83    68     2    gi_1
80    76    77    82     2    gi_1
77    74    80    81     2    gi_1
76    73    80    79     2    gi_1
74    70    77    78     2    gi_1
72    76    70    73     2    gi_1
72    69    73    75     2    gi_1
69    67    72    71     2    gi_1
67    74    73    70     2    gi_1
65    70    69    67     2    gi_1
64    62    66    65     2    gi_2
62    58    64    63     2    gi_1
```

2.2 Axinellin A

2.2.1 Manual

Parameters obtained for Axinellin A using only the manually specified Athenaeum. Only those residues with a preceding proline residue are not mapped.

```
[ atoms ]
1       N      1      TEST        N      1    -0.31000   14.0067
2       H      1      TEST        H      2     0.31000    1.0080
```

```
3    CH1    1    TEST    C    3     0.00000   13.0190
4    %%%    1    TEST    C    4     0.00000   14.0269  ; UNMAPPED
5    %%%    1    TEST    C    5     0.00000   12.0110  ; UNMAPPED
6    %%%    1    TEST    C    6     0.00000   12.0110  ; UNMAPPED
7    %%%    1    TEST    O    7     0.00000   15.9994  ; UNMAPPED
8    %%%    1    TEST    O    8     0.00000   15.9994  ; UNMAPPED
9    %%%    1    TEST    N    9     0.00000   14.0067  ; UNMAPPED
10    %%%    1    TEST    H    10     0.00000    1.0080  ; UNMAPPED
11    %%%    1    TEST    H    11     0.00000    1.0080  ; UNMAPPED
12      N    1    TEST    N    12     0.00000   14.0067
13   CH2r    1    TEST    C    13     0.00000   14.0269
14    CH1    1    TEST    C    14     0.00000   13.0190
15   CH2r    1    TEST    C    15     0.00000   14.0269
16   CH2r    1    TEST    C    16     0.00000   14.0269
17      C    1    TEST    C    17     0.45000   12.0110
18      O    1    TEST    O    18    -0.45000   15.9994
19      N    1    TEST    N    19    -0.31000   14.0067
20      H    1    TEST    H    20     0.31000    1.0080
21    CH1    1    TEST    C    21     0.00000   13.0190
22    CH2    1    TEST    C    22     0.00000   14.0269
23      C    1    TEST    C    23     0.45000   12.0110
24      C    1    TEST    C    24     0.00000   12.0110
25      O    1    TEST    O    25    -0.45000   15.9994
26      C    1    TEST    C    26    -0.14000   12.0110
27      C    1    TEST    C    27    -0.14000   12.0110
28     HC    1    TEST    H    28     0.14000    1.0080
29      C    1    TEST    C    29    -0.14000   12.0110
30      C    1    TEST    C    30    -0.14000   12.0110
31     HC    1    TEST    H    31     0.14000    1.0080
32     HC    1    TEST    H    32     0.14000    1.0080
33      C    1    TEST    C    33    -0.14000   12.0110
34     HC    1    TEST    H    34     0.14000    1.0080
35     HC    1    TEST    H    35     0.14000    1.0080
36      N    1    TEST    N    36    -0.31000   14.0067
37      H    1    TEST    H    37     0.31000    1.0080
38    CH1    1    TEST    C    38     0.00000   13.0190
39    CH1    1    TEST    C    39     0.26600   13.0190
40      C    1    TEST    C    40     0.45000   12.0110
41    CH3    1    TEST    C    41     0.00000   15.0349
42     OA    1    TEST    O    42    -0.67400   15.9994
43      O    1    TEST    O    43    -0.45000   15.9994
44      H    1    TEST    H    44     0.40800    1.0080
45      N    1    TEST    N    45    -0.31000   14.0067
46      H    1    TEST    H    46     0.31000    1.0080
47    CH1    1    TEST    C    47     0.00000   13.0190
```

```
48    CH1     1     TEST      C    48     0.00000   13.0190
49      C     1     TEST      C    49     0.45000   12.0110
50    CH3     1     TEST      C    50     0.00000   15.0349
51    CH2     1     TEST      C    51     0.00000   14.0269
52      O     1     TEST      O    52    -0.45000   15.9994
53    CH3     1     TEST      C    53     0.00000   15.0349
54      C     1     TEST      C    54     0.45000   12.0110
55    CH1     1     TEST      C    55     0.00000   13.0190
56      O     1     TEST      O    56    -0.45000   15.9994
57      N     1     TEST      N    57     0.00000   14.0067
58   CH2r     1     TEST      C    58     0.00000   14.0269
59   CH2r     1     TEST      C    59     0.00000   14.0269
60   CH2r     1     TEST      C    60     0.00000   14.0269
61    %%%     1     TEST      C    61     0.00000   12.0110   ; UNMAPPED
62    CH1     1     TEST      C    62     0.00000   13.0190
63    %%%     1     TEST      O    63     0.00000   15.9994   ; UNMAPPED
64      N     1     TEST      N    64    -0.31000   14.0067
65    %%%     1     TEST      C    65     0.00000   14.0269   ; UNMAPPED
66      H     1     TEST      H    66     0.31000    1.0080
67    %%%     1     TEST      C    67     0.00000   12.0110   ; UNMAPPED
68    %%%     1     TEST      C    68     0.00000   12.0110   ; UNMAPPED
69    %%%     1     TEST      C    69     0.00000   12.0110   ; UNMAPPED
70    %%%     1     TEST      H    70     0.00000    1.0080   ; UNMAPPED
71    %%%     1     TEST      C    71     0.00000   12.0110   ; UNMAPPED
72    %%%     1     TEST      C    72     0.00000   12.0110   ; UNMAPPED
73    %%%     1     TEST      H    73     0.00000    1.0080   ; UNMAPPED
74    %%%     1     TEST      H    74     0.00000    1.0080   ; UNMAPPED
75    %%%     1     TEST      C    75     0.00000   12.0110   ; UNMAPPED
76    %%%     1     TEST      H    76     0.00000    1.0080   ; UNMAPPED
77    %%%     1     TEST      H    77     0.00000    1.0080   ; UNMAPPED

[ bonds ]
3      4     2    gb_UNMAPPED
3      5     2    gb_UNMAPPED
4      6     2    gb_UNMAPPED
5      7     2    gb_UNMAPPED
6      8     2    gb_UNMAPPED
6      9     2    gb_UNMAPPED
9     10     2    gb_UNMAPPED
9     11     2    gb_UNMAPPED
61     62     2    gb_UNMAPPED
61     63     2    gb_UNMAPPED
62     65     2    gb_UNMAPPED
65     67     2    gb_UNMAPPED
67     68     2    gb_UNMAPPED
```

```
67    69    2    gb_UNMAPPED
68    70    2    gb_UNMAPPED
68    71    2    gb_UNMAPPED
69    72    2    gb_UNMAPPED
69    73    2    gb_UNMAPPED
71    74    2    gb_UNMAPPED
71    75    2    gb_UNMAPPED
72    75    2    gb_UNMAPPED
72    76    2    gb_UNMAPPED
75    77    2    gb_UNMAPPED
1    2    2    gb_2
1    3    2    gb_21
1    54    2    gb_10
5    12    2    gb_10
12    13    2    gb_21
12    14    2    gb_21
13    15    2    gb_27
14    16    2    gb_27
14    17    2    gb_27
15    16    2    gb_27
17    18    2    gb_5
17    19    2    gb_10
19    20    2    gb_2
19    21    2    gb_21
21    22    2    gb_27
21    23    2    gb_27
22    24    2    gb_27
23    25    2    gb_5
23    36    2    gb_10
24    26    2    gb_16
24    27    2    gb_16
26    28    2    gb_3
26    29    2    gb_16
27    30    2    gb_16
27    31    2    gb_3
29    32    2    gb_3
29    33    2    gb_16
30    33    2    gb_16
30    34    2    gb_3
33    35    2    gb_3
36    37    2    gb_2
36    38    2    gb_21
38    39    2    gb_27
38    40    2    gb_27
39    41    2    gb_27
```

```
39     42      2    gb_18
40     43      2    gb_5
40     45      2    gb_10
42     44      2    gb_1
45     46      2    gb_2
45     47      2    gb_21
47     48      2    gb_27
47     49      2    gb_27
48     50      2    gb_27
48     51      2    gb_27
49     52      2    gb_5
49     64      2    gb_10
51     53      2    gb_27
54     55      2    gb_27
54     56      2    gb_5
55     57      2    gb_21
55     58      2    gb_27
57     59      2    gb_21
57     61      2    gb_10
58     60      2    gb_27
59     60      2    gb_27
62     64      2    gb_21
64     66      2    gb_2

[ angles ]
1      3      4      2     ga_UNMAPPED
1      3      5      2     ga_UNMAPPED
4      3      5      2     ga_UNMAPPED
3      4      6      2     ga_UNMAPPED
3      5      7      2     ga_UNMAPPED
3      5     12      2     ga_UNMAPPED
7      5     12      2     ga_UNMAPPED
4      6      8      2     ga_UNMAPPED
4      6      9      2     ga_UNMAPPED
8      6      9      2     ga_UNMAPPED
6      9     10      2     ga_UNMAPPED
6      9     11      2     ga_UNMAPPED
10      9     11      2     ga_UNMAPPED
57     61     62      2     ga_UNMAPPED
57     61     63      2     ga_UNMAPPED
62     61     63      2     ga_UNMAPPED
61     62     64      2     ga_UNMAPPED
61     62     65      2     ga_UNMAPPED
64     62     65      2     ga_UNMAPPED
62     65     67      2     ga_UNMAPPED
```

65	67	68	2	ga_UNMAPPED
65	67	69	2	ga_UNMAPPED
68	67	69	2	ga_UNMAPPED
67	68	70	2	ga_UNMAPPED
67	68	71	2	ga_UNMAPPED
70	68	71	2	ga_UNMAPPED
67	69	72	2	ga_UNMAPPED
67	69	73	2	ga_UNMAPPED
72	69	73	2	ga_UNMAPPED
68	71	74	2	ga_UNMAPPED
68	71	75	2	ga_UNMAPPED
74	71	75	2	ga_UNMAPPED
69	72	75	2	ga_UNMAPPED
69	72	76	2	ga_UNMAPPED
75	72	76	2	ga_UNMAPPED
71	75	72	2	ga_UNMAPPED
71	75	77	2	ga_UNMAPPED
72	75	77	2	ga_UNMAPPED
2	1	3	2	ga_18
2	1	54	2	ga_32
3	1	54	2	ga_31
5	12	13	2	ga_31
5	12	14	2	ga_31
13	12	14	2	ga_21
12	13	15	2	ga_13
12	14	16	2	ga_13
12	14	17	2	ga_13
16	14	17	2	ga_13
13	15	16	2	ga_13
14	16	15	2	ga_13
14	17	18	2	ga_30
14	17	19	2	ga_19
18	17	19	2	ga_33
17	19	20	2	ga_32
17	19	21	2	ga_31
20	19	21	2	ga_18
19	21	22	2	ga_13
19	21	23	2	ga_13
22	21	23	2	ga_13
21	22	24	2	ga_15
21	23	25	2	ga_30
21	23	36	2	ga_19
25	23	36	2	ga_33
22	24	26	2	ga_27
22	24	27	2	ga_27

26	24	27	2	ga_27
24	26	28	2	ga_25
24	26	29	2	ga_27
28	26	29	2	ga_25
24	27	30	2	ga_27
24	27	31	2	ga_25
30	27	31	2	ga_25
26	29	32	2	ga_25
26	29	33	2	ga_27
32	29	33	2	ga_25
27	30	33	2	ga_27
27	30	34	2	ga_25
33	30	34	2	ga_25
29	33	30	2	ga_27
29	33	35	2	ga_25
30	33	35	2	ga_25
23	36	37	2	ga_32
23	36	38	2	ga_31
37	36	38	2	ga_18
36	38	39	2	ga_13
36	38	40	2	ga_13
39	38	40	2	ga_13
38	39	41	2	ga_15
38	39	42	2	ga_13
41	39	42	2	ga_15
38	40	43	2	ga_30
38	40	45	2	ga_19
43	40	45	2	ga_33
39	42	44	2	ga_12
40	45	46	2	ga_32
40	45	47	2	ga_31
46	45	47	2	ga_18
45	47	48	2	ga_13
45	47	49	2	ga_13
48	47	49	2	ga_13
47	48	50	2	ga_15
47	48	51	2	ga_15
50	48	51	2	ga_15
47	49	52	2	ga_30
47	49	64	2	ga_19
52	49	64	2	ga_33
48	51	53	2	ga_15
1	54	55	2	ga_19
1	54	56	2	ga_33
55	54	56	2	ga_30

```
54     55     57      2    ga_13
54     55     58      2    ga_13
57     55     58      2    ga_13
55     57     59      2    ga_21
55     57     61      2    ga_31
59     57     61      2    ga_31
55     58     60      2    ga_13
57     59     60      2    ga_13
58     60     59      2    ga_13
49     64     62      2    ga_31
49     64     66      2    ga_32
62     64     66      2    ga_18

[ dihedrals ]
2      1      3      4           UNMAPPED
1      3      4      6           UNMAPPED
1      3      5      7           UNMAPPED
3      4      6      8           UNMAPPED
3      5     12     13           UNMAPPED
4      6      9     10           UNMAPPED
55     57     61     62            UNMAPPED
57     61     62     64            UNMAPPED
61     62     64     49            UNMAPPED
61     62     65     67            UNMAPPED
62     65     67     68            UNMAPPED
65     67     68     70            UNMAPPED
65     67     69     72            UNMAPPED
67     68     71     74            UNMAPPED
67     69     72     75            UNMAPPED
68     71     75     72            UNMAPPED
69     72     75     71            UNMAPPED
3      1     54     55      1    gd_14
14     12     13     15      1    gd_39
5     12     14     17      1    gd_43
5     12     14     17      1    gd_44
12     13     15     16      1    gd_34
12     14     16     15      1    gd_34
12     14     17     19      1    gd_42
12     14     17     19      1    gd_45
13     15     16     14      1    gd_34
14     17     19     21      1    gd_14
17     19     21     23      1    gd_43
17     19     21     23      1    gd_44
19     21     22     24      1    gd_34
19     21     23     36      1    gd_42
```

19	21	23	36	1	gd_45
21	22	24	26	1	gd_40
21	22	24	27	1	gd_40
21	23	36	38	1	gd_14
27	24	26	29	2	gi_1
26	24	27	30	2	gi_1
24	26	29	33	2	gi_1
24	27	30	33	2	gi_1
26	29	33	30	2	gi_1
27	30	33	29	2	gi_1
23	36	38	40	1	gd_43
23	36	38	40	1	gd_44
36	38	39	42	1	gd_34
36	38	40	45	1	gd_42
36	38	40	45	1	gd_45
38	39	42	44	1	gd_23
38	40	45	47	1	gd_14
40	45	47	49	1	gd_43
40	45	47	49	1	gd_44
45	47	48	51	1	gd_34
45	47	49	64	1	gd_42
45	47	49	64	1	gd_45
47	48	51	53	1	gd_34
47	49	64	62	1	gd_14
1	54	55	57	1	gd_42
1	54	55	57	1	gd_45
54	55	57	61	1	gd_43
54	55	57	61	1	gd_44
57	55	58	60	1	gd_34
55	57	59	60	1	gd_39
55	58	60	59	1	gd_34
57	59	60	58	1	gd_34
40	38	45	43	2	gi_1
38	41	42	39	2	gi_2
38	36	40	39	2	gi_2
36	23	38	37	2	gi_1
18	19	14	17	2	gi_1
14	12	17	16	2	gi_2
12	5	14	13	2	gi_1
54	55	1	56	2	gi_1
55	57	54	58	2	gi_2
57	61	55	59	2	gi_1
23	21	36	25	2	gi_1
35	30	29	33	2	gi_1
34	27	33	30	2	gi_1

```
29    33    26    32     2    gi_1
27    24    30    31     2    gi_1
26    24    29    28     2    gi_1
22    27    26    24     2    gi_1
21    19    23    22     2    gi_2
19    17    21    20     2    gi_1
35    29    30    33     2    gi_1
22    26    27    24     2    gi_1
49    47    64    52     2    gi_1
47    50    51    48     2    gi_2
47    45    49    48     2    gi_2
45    40    47    46     2    gi_1
64    49    62    66     2    gi_1
 2     3    54     1     2    gi_1
```

2.2.2 Automatic

Parameters obtained for Axinellin A using first the manually specified Athenaeum then the automatically generated Athenaeum. Only two dihedral terms involving the proline residues have not been mapped successfully.

```
[ atoms ]
1       N     1     TEST       N     1    -0.31000   14.0067
2       H     1     TEST       H     2     0.31000    1.0080
3     CH1     1     TEST       C     3     0.00000   13.0190
4     CH2     1     TEST       C     4     0.00000   14.0269
5       C     1     TEST       C     5     0.45000   12.0110
6       C     1     TEST       C     6     0.29000   12.0110
7       O     1     TEST       O     7    -0.45000   15.9994
8       O     1     TEST       O     8    -0.45000   15.9994
9      NT     1     TEST       N     9    -0.72000   14.0067
10      H     1     TEST       H    10     0.44000    1.0080
11      H     1     TEST       H    11     0.44000    1.0080
12      N     1     TEST       N    12     0.00000   14.0067
13   CH2r     1     TEST       C    13     0.00000   14.0269
14    CH1     1     TEST       C    14     0.00000   13.0190
15   CH2r     1     TEST       C    15     0.00000   14.0269
16   CH2r     1     TEST       C    16     0.00000   14.0269
17      C     1     TEST       C    17     0.45000   12.0110
18      O     1     TEST       O    18    -0.45000   15.9994
19      N     1     TEST       N    19    -0.31000   14.0067
20      H     1     TEST       H    20     0.31000    1.0080
21    CH1     1     TEST       C    21     0.00000   13.0190
22    CH2     1     TEST       C    22     0.00000   14.0269
23      C     1     TEST       C    23     0.45000   12.0110
24      C     1     TEST       C    24     0.00000   12.0110
```

25	O	1	TEST	O	25	-0.45000	15.9994
26	C	1	TEST	C	26	-0.14000	12.0110
27	C	1	TEST	C	27	-0.14000	12.0110
28	HC	1	TEST	H	28	0.14000	1.0080
29	C	1	TEST	C	29	-0.14000	12.0110
30	C	1	TEST	C	30	-0.14000	12.0110
31	HC	1	TEST	H	31	0.14000	1.0080
32	HC	1	TEST	H	32	0.14000	1.0080
33	C	1	TEST	C	33	-0.14000	12.0110
34	HC	1	TEST	H	34	0.14000	1.0080
35	HC	1	TEST	H	35	0.14000	1.0080
36	N	1	TEST	N	36	-0.31000	14.0067
37	H	1	TEST	H	37	0.31000	1.0080
38	CH1	1	TEST	C	38	0.00000	13.0190
39	CH1	1	TEST	C	39	0.26600	13.0190
40	C	1	TEST	C	40	0.45000	12.0110
41	CH3	1	TEST	C	41	0.00000	15.0349
42	OA	1	TEST	O	42	-0.67400	15.9994
43	O	1	TEST	O	43	-0.45000	15.9994
44	H	1	TEST	H	44	0.40800	1.0080
45	N	1	TEST	N	45	-0.31000	14.0067
46	H	1	TEST	H	46	0.31000	1.0080
47	CH1	1	TEST	C	47	0.00000	13.0190
48	CH1	1	TEST	C	48	0.00000	13.0190
49	C	1	TEST	C	49	0.45000	12.0110
50	CH3	1	TEST	C	50	0.00000	15.0349
51	CH2	1	TEST	C	51	0.00000	14.0269
52	O	1	TEST	O	52	-0.45000	15.9994
53	CH3	1	TEST	C	53	0.00000	15.0349
54	C	1	TEST	C	54	0.45000	12.0110
55	CH1	1	TEST	C	55	0.00000	13.0190
56	O	1	TEST	O	56	-0.45000	15.9994
57	N	1	TEST	N	57	0.00000	14.0067
58	CH2r	1	TEST	C	58	0.00000	14.0269
59	CH2r	1	TEST	C	59	0.00000	14.0269
60	CH2r	1	TEST	C	60	0.00000	14.0269
61	C	1	TEST	C	61	0.45000	12.0110
62	CH1	1	TEST	C	62	0.00000	13.0190
63	O	1	TEST	O	63	-0.45000	15.9994
64	N	1	TEST	N	64	-0.31000	14.0067
65	CH2	1	TEST	C	65	0.00000	14.0269
66	H	1	TEST	H	66	0.31000	1.0080
67	C	1	TEST	C	67	0.00000	12.0110
68	C	1	TEST	C	68	-0.14000	12.0110
69	C	1	TEST	C	69	-0.14000	12.0110

```
70      HC      1      TEST        H     70      0.14000    1.0080
71       C      1      TEST        C     71     -0.14000   12.0110
72       C      1      TEST        C     72     -0.14000   12.0110
73      HC      1      TEST        H     73      0.14000    1.0080
74      HC      1      TEST        H     74      0.14000    1.0080
75       C      1      TEST        C     75     -0.14000   12.0110
76      HC      1      TEST        H     76      0.14000    1.0080
77      HC      1      TEST        H     77      0.14000    1.0080

[ bonds ]
1      2      2    gb_2
1      3      2    gb_21
1     54      2    gb_10
3      4      2    gb_27
3      5      2    gb_27
4      6      2    gb_27
5      7      2    gb_5
5     12      2    gb_10
6      8      2    gb_5
6      9      2    gb_9
9     10      2    gb_2
9     11      2    gb_2
12     13      2    gb_21
12     14      2    gb_21
13     15      2    gb_27
14     16      2    gb_27
14     17      2    gb_27
15     16      2    gb_27
17     18      2    gb_5
17     19      2    gb_10
19     20      2    gb_2
19     21      2    gb_21
21     22      2    gb_27
21     23      2    gb_27
22     24      2    gb_27
23     25      2    gb_5
23     36      2    gb_10
24     26      2    gb_16
24     27      2    gb_16
26     28      2    gb_3
26     29      2    gb_16
27     30      2    gb_16
27     31      2    gb_3
29     32      2    gb_3
29     33      2    gb_16
```

30	33	2	gb_16
30	34	2	gb_3
33	35	2	gb_3
36	37	2	gb_2
36	38	2	gb_21
38	39	2	gb_27
38	40	2	gb_27
39	41	2	gb_27
39	42	2	gb_18
40	43	2	gb_5
40	45	2	gb_10
42	44	2	gb_1
45	46	2	gb_2
45	47	2	gb_21
47	48	2	gb_27
47	49	2	gb_27
48	50	2	gb_27
48	51	2	gb_27
49	52	2	gb_5
49	64	2	gb_10
51	53	2	gb_27
54	55	2	gb_27
54	56	2	gb_5
55	57	2	gb_21
55	58	2	gb_27
57	59	2	gb_21
57	61	2	gb_10
58	60	2	gb_27
59	60	2	gb_27
61	62	2	gb_27
61	63	2	gb_5
62	64	2	gb_21
62	65	2	gb_27
64	66	2	gb_2
65	67	2	gb_27
67	68	2	gb_16
67	69	2	gb_16
68	70	2	gb_3
68	71	2	gb_16
69	72	2	gb_16
69	73	2	gb_3
71	74	2	gb_3
71	75	2	gb_16
72	75	2	gb_16
72	76	2	gb_3

```
75    77     2    gb_3

[ angles ]
2     1     3     2    ga_18
2     1    54     2    ga_32
3     1    54     2    ga_31
1     3     4     2    ga_13
1     3     5     2    ga_13
4     3     5     2    ga_13
3     4     6     2    ga_15
3     5     7     2    ga_30
3     5    12     2    ga_19
7     5    12     2    ga_33
4     6     8     2    ga_30
4     6     9     2    ga_19
8     6     9     2    ga_33
6     9    10     2    ga_23
6     9    11     2    ga_23
10     9    11     2    ga_24
5    12    13     2    ga_31
5    12    14     2    ga_31
13    12    14     2    ga_21
12    13    15     2    ga_13
12    14    16     2    ga_13
12    14    17     2    ga_13
16    14    17     2    ga_13
13    15    16     2    ga_13
14    16    15     2    ga_13
14    17    18     2    ga_30
14    17    19     2    ga_19
18    17    19     2    ga_33
17    19    20     2    ga_32
17    19    21     2    ga_31
20    19    21     2    ga_18
19    21    22     2    ga_13
19    21    23     2    ga_13
22    21    23     2    ga_13
21    22    24     2    ga_15
21    23    25     2    ga_30
21    23    36     2    ga_19
25    23    36     2    ga_33
22    24    26     2    ga_27
22    24    27     2    ga_27
26    24    27     2    ga_27
24    26    28     2    ga_25
```

24	26	29	2	ga_27
28	26	29	2	ga_25
24	27	30	2	ga_27
24	27	31	2	ga_25
30	27	31	2	ga_25
26	29	32	2	ga_25
26	29	33	2	ga_27
32	29	33	2	ga_25
27	30	33	2	ga_27
27	30	34	2	ga_25
33	30	34	2	ga_25
29	33	30	2	ga_27
29	33	35	2	ga_25
30	33	35	2	ga_25
23	36	37	2	ga_32
23	36	38	2	ga_31
37	36	38	2	ga_18
36	38	39	2	ga_13
36	38	40	2	ga_13
39	38	40	2	ga_13
38	39	41	2	ga_15
38	39	42	2	ga_13
41	39	42	2	ga_15
38	40	43	2	ga_30
38	40	45	2	ga_19
43	40	45	2	ga_33
39	42	44	2	ga_12
40	45	46	2	ga_32
40	45	47	2	ga_31
46	45	47	2	ga_18
45	47	48	2	ga_13
45	47	49	2	ga_13
48	47	49	2	ga_13
47	48	50	2	ga_15
47	48	51	2	ga_15
50	48	51	2	ga_15
47	49	52	2	ga_30
47	49	64	2	ga_19
52	49	64	2	ga_33
48	51	53	2	ga_15
1	54	55	2	ga_19
1	54	56	2	ga_33
55	54	56	2	ga_30
54	55	57	2	ga_13
54	55	58	2	ga_13

```
57     55     58      2    ga_13
55     57     59      2    ga_21
55     57     61      2    ga_31
59     57     61      2    ga_31
55     58     60      2    ga_13
57     59     60      2    ga_13
58     60     59      2    ga_13
57     61     62      2    ga_19
57     61     63      2    ga_33
62     61     63      2    ga_30
61     62     64      2    ga_13
61     62     65      2    ga_13
64     62     65      2    ga_13
49     64     62      2    ga_31
49     64     66      2    ga_32
62     64     66      2    ga_18
62     65     67      2    ga_15
65     67     68      2    ga_27
65     67     69      2    ga_27
68     67     69      2    ga_27
67     68     70      2    ga_25
67     68     71      2    ga_27
70     68     71      2    ga_25
67     69     72      2    ga_27
67     69     73      2    ga_25
72     69     73      2    ga_25
68     71     74      2    ga_25
68     71     75      2    ga_27
74     71     75      2    ga_25
69     72     75      2    ga_27
69     72     76      2    ga_25
75     72     76      2    ga_25
71     75     72      2    ga_27
71     75     77      2    ga_25
72     75     77      2    ga_25

[ dihedrals ]
1      3      5      7           UNMAPPED
57     61     62     64           UNMAPPED
54      1      3      5      1    gd_43
54      1      3      5      1    gd_44
3      1     54     55      1    gd_14
1      3      4      6      1    gd_34
3      4      6      9      1    gd_40
3      5     12     14      1    gd_14
```

4	6	9	10	1	gd_14
14	12	13	15	1	gd_39
5	12	14	17	1	gd_43
5	12	14	17	1	gd_44
12	13	15	16	1	gd_34
12	14	16	15	1	gd_34
12	14	17	19	1	gd_42
12	14	17	19	1	gd_45
13	15	16	14	1	gd_34
14	17	19	21	1	gd_14
17	19	21	23	1	gd_43
17	19	21	23	1	gd_44
19	21	22	24	1	gd_34
19	21	23	36	1	gd_42
19	21	23	36	1	gd_45
21	22	24	26	1	gd_40
21	22	24	27	1	gd_40
21	23	36	38	1	gd_14
27	24	26	29	2	gi_1
26	24	27	30	2	gi_1
24	26	29	33	2	gi_1
24	27	30	33	2	gi_1
26	29	33	30	2	gi_1
27	30	33	29	2	gi_1
23	36	38	40	1	gd_43
23	36	38	40	1	gd_44
36	38	39	42	1	gd_34
36	38	40	45	1	gd_42
36	38	40	45	1	gd_45
38	39	42	44	1	gd_23
38	40	45	47	1	gd_14
40	45	47	49	1	gd_43
40	45	47	49	1	gd_44
45	47	48	51	1	gd_34
45	47	49	64	1	gd_42
45	47	49	64	1	gd_45
47	48	51	53	1	gd_34
47	49	64	62	1	gd_14
1	54	55	57	1	gd_42
1	54	55	57	1	gd_45
54	55	57	61	1	gd_43
54	55	57	61	1	gd_44
57	55	58	60	1	gd_34
55	57	59	60	1	gd_39
55	57	61	62	1	gd_14

55	58	60	59	1	gd_34
57	59	60	58	1	gd_34
61	62	64	49	1	gd_43
61	62	64	49	1	gd_44
64	62	65	67	1	gd_34
62	65	67	68	1	gd_40
62	65	67	69	1	gd_40
69	67	68	71	2	gi_1
68	67	69	72	2	gi_1
67	68	71	75	2	gi_1
67	69	72	75	2	gi_1
68	71	75	72	2	gi_1
69	72	75	71	2	gi_1
18	19	14	17	2	gi_1
14	12	17	16	2	gi_2
12	5	14	13	2	gi_1
54	55	1	56	2	gi_1
55	57	54	58	2	gi_2
57	61	55	59	2	gi_1
40	38	45	43	2	gi_1
38	41	42	39	2	gi_2
38	36	40	39	2	gi_2
36	23	38	37	2	gi_1
23	21	36	25	2	gi_1
35	30	29	33	2	gi_1
34	27	33	30	2	gi_1
29	33	26	32	2	gi_1
27	24	30	31	2	gi_1
26	24	29	28	2	gi_1
22	27	26	24	2	gi_1
21	19	23	22	2	gi_2
19	17	21	20	2	gi_1
35	29	30	33	2	gi_1
22	26	27	24	2	gi_1
49	47	64	52	2	gi_1
47	50	51	48	2	gi_2
47	45	49	48	2	gi_2
45	40	47	46	2	gi_1
64	49	62	66	2	gi_1
1	54	3	2	2	gi_1
62	64	61	65	2	gi_2
3	1	5	4	2	gi_2
6	11	10	9	2	gi_1
4	9	8	6	2	gi_1
6	10	11	9	2	gi_1

```
34     33     27     30      2    gi_1
29     26     33     32      2    gi_1
75     72     71     77      2    gi_1
71     75     68     74      2    gi_1
75     71     72     77      2    gi_1
72     75     69     76      2    gi_1
72     69     75     76      2    gi_1
71     68     75     74      2    gi_1
68     67     71     70      2    gi_1
69     67     72     73      2    gi_1
65     68     69     67      2    gi_1
65     69     68     67      2    gi_1
5     3    12     7     2    gi_1
61     62     57     63      2    gi_1
```

2.3 Polymyxin B3

2.3.1 Manual

Parameters obtained for Polymyxin B3 using only the manually specified Athenaeum. The parts unmapped are the DAB residues.

```
[ atoms ]
1    %%%     1     TEST       C     1     0.00000   12.0110  ; UNMAPPED
2    %%%     1     TEST       O     2     0.00000   15.9994  ; UNMAPPED
3    %%%     1     TEST      CA     3     0.00000   14.0269  ; UNMAPPED
4    CH2     1     TEST      CB     4     0.00000   14.0269
5    CH2     1     TEST      CC     5     0.00000   14.0269
6    CH2     1     TEST      CD     6     0.00000   14.0269
7    CH2     1     TEST      CE     7     0.00000   14.0269
8    CH2     1     TEST      CG     8     0.00000   14.0269
9    CH3     1     TEST      CH     9     0.00000   15.0349
10     %%%     1     TEST       N     10     0.00000   14.0067  ; UNMAPPED
11     %%%     1     TEST       H     11     0.00000    1.0080  ; UNMAPPED
12     CH1     1     TEST      CA     12     0.00000   13.0190
13     %%%     1     TEST      CB     13     0.00000   14.0269  ; UNMAPPED
14     %%%     1     TEST      CG     14     0.00000   14.0269  ; UNMAPPED
15     %%%     1     TEST      ND     15     0.00000   14.0067  ; UNMAPPED
16     %%%     1     TEST     HD1     16     0.00000    1.0080  ; UNMAPPED
17     %%%     1     TEST     HD2     17     0.00000    1.0080  ; UNMAPPED
18     %%%     1     TEST     HD3     18     0.00000    1.0080  ; UNMAPPED
19       C     1     TEST       C     19     0.45000   12.0110
20       O     1     TEST       O     20    -0.45000   15.9994
21       N     1     TEST       N     21    -0.31000   14.0067
22       H     1     TEST       H     22     0.31000    1.0080
23     CH1     1     TEST      CA     23     0.00000   13.0190
24     CH1     1     TEST      CB     24     0.26600   13.0190
```

25	OA	1	TEST	OG1	25	-0.67400	15.9994	
26	H	1	TEST	HG1	26	0.40800	1.0080	
27	CH3	1	TEST	CG2	27	0.00000	15.0349	
28	C	1	TEST	C	28	0.45000	12.0110	
29	O	1	TEST	O	29	-0.45000	15.9994	
30	N	1	TEST	N	30	-0.31000	14.0067	
31	H	1	TEST	H	31	0.31000	1.0080	
32	CH1	1	TEST	CA	32	0.00000	13.0190	
33	%%%	1	TEST	CB	33	0.00000	14.0269	; UNMAPPED
34	%%%	1	TEST	CG	34	0.00000	14.0269	; UNMAPPED
35	%%%	1	TEST	ND	35	0.00000	14.0067	; UNMAPPED
36	%%%	1	TEST	HD1	36	0.00000	1.0080	; UNMAPPED
37	%%%	1	TEST	HD2	37	0.00000	1.0080	; UNMAPPED
38	%%%	1	TEST	HD3	38	0.00000	1.0080	; UNMAPPED
39	C	1	TEST	C	39	0.45000	12.0110	
40	O	1	TEST	O	40	-0.45000	15.9994	
41	N	1	TEST	N	41	-0.31000	14.0067	
42	H	1	TEST	H	42	0.31000	1.0080	
43	CH1	1	TEST	CA	43	0.00000	13.0190	
44	%%%	1	TEST	CB	44	0.00000	14.0269	; UNMAPPED
45	%%%	1	TEST	CG	45	0.00000	14.0269	; UNMAPPED
46	%%%	1	TEST	ND	46	0.00000	14.0067	; UNMAPPED
47	%%%	1	TEST	HD1	47	0.00000	1.0080	; UNMAPPED
48	C	1	TEST	C	48	0.45000	12.0110	
49	O	1	TEST	O	49	-0.45000	15.9994	
50	N	1	TEST	N	50	-0.31000	14.0067	
51	H	1	TEST	H	51	0.31000	1.0080	
52	CH1	1	TEST	CA	52	0.00000	13.0190	
53	%%%	1	TEST	CB	53	0.00000	14.0269	; UNMAPPED
54	%%%	1	TEST	CG	54	0.00000	14.0269	; UNMAPPED
55	%%%	1	TEST	ND	55	0.00000	14.0067	; UNMAPPED
56	%%%	1	TEST	HD1	56	0.00000	1.0080	; UNMAPPED
57	%%%	1	TEST	HD2	57	0.00000	1.0080	; UNMAPPED
58	%%%	1	TEST	HD3	58	0.00000	1.0080	; UNMAPPED
59	C	1	TEST	C	59	0.45000	12.0110	
60	O	1	TEST	O	60	-0.45000	15.9994	
61	N	1	TEST	N	61	-0.31000	14.0067	
62	H	1	TEST	H	62	0.31000	1.0080	
63	CH1	1	TEST	CA	63	0.00000	13.0190	
64	CH2	1	TEST	CB	64	0.00000	14.0269	
65	C	1	TEST	CG	65	0.00000	12.0110	
66	C	1	TEST	CD1	66	-0.14000	12.0110	
67	HC	1	TEST	HD1	67	0.14000	1.0080	
68	C	1	TEST	CD2	68	-0.14000	12.0110	
69	HC	1	TEST	HD2	69	0.14000	1.0080	

70	C	1	TEST	CE1	70	-0.14000	12.0110	
71	HC	1	TEST	HE1	71	0.14000	1.0080	
72	C	1	TEST	CE2	72	-0.14000	12.0110	
73	HC	1	TEST	HE2	73	0.14000	1.0080	
74	C	1	TEST	CZ	74	-0.14000	12.0110	
75	HC	1	TEST	HZ	75	0.14000	1.0080	
76	C	1	TEST	C	76	0.45000	12.0110	
77	O	1	TEST	O	77	-0.45000	15.9994	
78	N	1	TEST	N	78	-0.31000	14.0067	
79	H	1	TEST	H	79	0.31000	1.0080	
80	CH1	1	TEST	CA	80	0.00000	13.0190	
81	CH2	1	TEST	CB	81	0.00000	14.0269	
82	CH1	1	TEST	CG	82	0.00000	13.0190	
83	CH3	1	TEST	CD1	83	0.00000	15.0349	
84	CH3	1	TEST	CD2	84	0.00000	15.0349	
85	C	1	TEST	C	85	0.45000	12.0110	
86	O	1	TEST	O	86	-0.45000	15.9994	
87	N	1	TEST	N	87	-0.31000	14.0067	
88	H	1	TEST	H	88	0.31000	1.0080	
89	CH1	1	TEST	CA	89	0.00000	13.0190	
90	%%%	1	TEST	CB	90	0.00000	14.0269	; UNMAPPED
91	%%%	1	TEST	CG	91	0.00000	14.0269	; UNMAPPED
92	%%%	1	TEST	ND	92	0.00000	14.0067	; UNMAPPED
93	%%%	1	TEST	HD1	93	0.00000	1.0080	; UNMAPPED
94	%%%	1	TEST	HD2	94	0.00000	1.0080	; UNMAPPED
95	%%%	1	TEST	HD3	95	0.00000	1.0080	; UNMAPPED
96	C	1	TEST	C	96	0.45000	12.0110	
97	O	1	TEST	O	97	-0.45000	15.9994	
98	N	1	TEST	N	98	-0.31000	14.0067	
99	H	1	TEST	H	99	0.31000	1.0080	
100	CH1	1	TEST	CA	100	0.00000	13.0190	
101	%%%	1	TEST	CB	101	0.00000	14.0269	; UNMAPPED
102	%%%	1	TEST	CG	102	0.00000	14.0269	; UNMAPPED
103	%%%	1	TEST	ND	103	0.00000	14.0067	; UNMAPPED
104	%%%	1	TEST	HD1	104	0.00000	1.0080	; UNMAPPED
105	%%%	1	TEST	HD2	105	0.00000	1.0080	; UNMAPPED
106	%%%	1	TEST	HD3	106	0.00000	1.0080	; UNMAPPED
107	C	1	TEST	C	107	0.45000	12.0110	
108	O	1	TEST	O	108	-0.45000	15.9994	
109	N	1	TEST	N	109	-0.31000	14.0067	
110	H	1	TEST	H	110	0.31000	1.0080	
111	CH1	1	TEST	CA	111	0.00000	13.0190	
112	CH1	1	TEST	CB	112	0.26600	13.0190	
113	OA	1	TEST	OG1	113	-0.67400	15.9994	
114	H	1	TEST	HG1	114	0.40800	1.0080	

```
115    CH3     1     TEST     CG2   115     0.00000   15.0349
116      C     1     TEST       C   116     0.45000   12.0110
117      O     1     TEST       O   117    -0.45000   15.9994

[ bonds ]
1     2     2    gb_UNMAPPED
1     3     2    gb_UNMAPPED
1    10     2    gb_UNMAPPED
10    11     2    gb_UNMAPPED
10    12     2    gb_UNMAPPED
12    13     2    gb_UNMAPPED
13    14     2    gb_UNMAPPED
14    15     2    gb_UNMAPPED
15    16     2    gb_UNMAPPED
15    17     2    gb_UNMAPPED
15    18     2    gb_UNMAPPED
32    33     2    gb_UNMAPPED
33    34     2    gb_UNMAPPED
34    35     2    gb_UNMAPPED
35    37     2    gb_UNMAPPED
35    36     2    gb_UNMAPPED
35    38     2    gb_UNMAPPED
43    44     2    gb_UNMAPPED
44    45     2    gb_UNMAPPED
45    46     2    gb_UNMAPPED
46    47     2    gb_UNMAPPED
52    53     2    gb_UNMAPPED
53    54     2    gb_UNMAPPED
54    55     2    gb_UNMAPPED
55    56     2    gb_UNMAPPED
55    57     2    gb_UNMAPPED
55    58     2    gb_UNMAPPED
89    90     2    gb_UNMAPPED
90    91     2    gb_UNMAPPED
91    92     2    gb_UNMAPPED
92    93     2    gb_UNMAPPED
92    94     2    gb_UNMAPPED
92    95     2    gb_UNMAPPED
100    101     2    gb_UNMAPPED
101    102     2    gb_UNMAPPED
102    103     2    gb_UNMAPPED
103    104     2    gb_UNMAPPED
103    105     2    gb_UNMAPPED
103    106     2    gb_UNMAPPED
3     4     2    gb_27
```

4	5	2	gb_27
5	6	2	gb_27
6	7	2	gb_27
7	8	2	gb_27
8	9	2	gb_27
12	19	2	gb_27
19	20	2	gb_5
19	21	2	gb_10
21	22	2	gb_2
21	23	2	gb_21
23	24	2	gb_27
23	28	2	gb_27
24	27	2	gb_27
24	25	2	gb_18
25	26	2	gb_1
28	30	2	gb_10
28	29	2	gb_5
30	31	2	gb_2
30	32	2	gb_21
32	39	2	gb_27
39	41	2	gb_10
39	40	2	gb_5
41	42	2	gb_2
41	43	2	gb_21
43	48	2	gb_27
46	116	2	gb_10
48	49	2	gb_5
48	50	2	gb_10
50	51	2	gb_2
50	52	2	gb_21
52	59	2	gb_27
59	60	2	gb_5
59	61	2	gb_10
61	62	2	gb_2
61	63	2	gb_21
63	76	2	gb_27
63	64	2	gb_27
64	65	2	gb_27
65	66	2	gb_16
65	68	2	gb_16
66	67	2	gb_3
66	70	2	gb_16
68	69	2	gb_3
68	72	2	gb_16
70	71	2	gb_3

```
70    74     2    gb_16
72    73     2    gb_3
72    74     2    gb_16
74    75     2    gb_3
76    77     2    gb_5
76    78     2    gb_10
78    79     2    gb_2
78    80     2    gb_21
80    85     2    gb_27
80    81     2    gb_27
81    82     2    gb_27
82    83     2    gb_27
82    84     2    gb_27
85    87     2    gb_10
85    86     2    gb_5
87    88     2    gb_2
87    89     2    gb_21
89    96     2    gb_27
96    98     2    gb_10
96    97     2    gb_5
98   100     2    gb_21
98    99     2    gb_2
100   107     2    gb_27
107   109     2    gb_10
107   108     2    gb_5
109   110     2    gb_2
109   111     2    gb_21
111   116     2    gb_27
111   112     2    gb_27
112   113     2    gb_18
112   115     2    gb_27
113   114     2    gb_1
116   117     2    gb_5

[ angles ]
2     1     3     2    ga_UNMAPPED
2     1    10     2    ga_UNMAPPED
3     1    10     2    ga_UNMAPPED
1     3     4     2    ga_UNMAPPED
1    10    11     2    ga_UNMAPPED
1    10    12     2    ga_UNMAPPED
11     10     12     2    ga_UNMAPPED
10     12     13     2    ga_UNMAPPED
10     12     19     2    ga_UNMAPPED
13     12     19     2    ga_UNMAPPED
```

12	13	14	2	ga_UNMAPPED
13	14	15	2	ga_UNMAPPED
14	15	16	2	ga_UNMAPPED
14	15	17	2	ga_UNMAPPED
14	15	18	2	ga_UNMAPPED
16	15	17	2	ga_UNMAPPED
16	15	18	2	ga_UNMAPPED
17	15	18	2	ga_UNMAPPED
30	32	39	2	ga_UNMAPPED
30	32	33	2	ga_UNMAPPED
39	32	33	2	ga_UNMAPPED
32	33	34	2	ga_UNMAPPED
33	34	35	2	ga_UNMAPPED
34	35	37	2	ga_UNMAPPED
34	35	36	2	ga_UNMAPPED
34	35	38	2	ga_UNMAPPED
37	35	36	2	ga_UNMAPPED
37	35	38	2	ga_UNMAPPED
36	35	38	2	ga_UNMAPPED
41	43	44	2	ga_UNMAPPED
41	43	48	2	ga_UNMAPPED
44	43	48	2	ga_UNMAPPED
43	44	45	2	ga_UNMAPPED
44	45	46	2	ga_UNMAPPED
45	46	47	2	ga_UNMAPPED
45	46	116	2	ga_UNMAPPED
47	46	116	2	ga_UNMAPPED
50	52	53	2	ga_UNMAPPED
50	52	59	2	ga_UNMAPPED
53	52	59	2	ga_UNMAPPED
52	53	54	2	ga_UNMAPPED
53	54	55	2	ga_UNMAPPED
54	55	56	2	ga_UNMAPPED
54	55	57	2	ga_UNMAPPED
54	55	58	2	ga_UNMAPPED
56	55	57	2	ga_UNMAPPED
56	55	58	2	ga_UNMAPPED
57	55	58	2	ga_UNMAPPED
87	89	90	2	ga_UNMAPPED
87	89	96	2	ga_UNMAPPED
90	89	96	2	ga_UNMAPPED
89	90	91	2	ga_UNMAPPED
90	91	92	2	ga_UNMAPPED
91	92	93	2	ga_UNMAPPED
91	92	94	2	ga_UNMAPPED

91	92	95	2	ga_UNMAPPED
93	92	94	2	ga_UNMAPPED
93	92	95	2	ga_UNMAPPED
94	92	95	2	ga_UNMAPPED
98	100	107	2	ga_UNMAPPED
98	100	101	2	ga_UNMAPPED
107	100	101	2	ga_UNMAPPED
100	101	102	2	ga_UNMAPPED
101	102	103	2	ga_UNMAPPED
102	103	104	2	ga_UNMAPPED
102	103	105	2	ga_UNMAPPED
102	103	106	2	ga_UNMAPPED
104	103	105	2	ga_UNMAPPED
104	103	106	2	ga_UNMAPPED
105	103	106	2	ga_UNMAPPED
3	4	5	2	ga_15
4	5	6	2	ga_15
5	6	7	2	ga_15
6	7	8	2	ga_15
7	8	9	2	ga_15
12	19	20	2	ga_30
12	19	21	2	ga_19
20	19	21	2	ga_33
19	21	22	2	ga_32
19	21	23	2	ga_31
22	21	23	2	ga_18
21	23	24	2	ga_13
21	23	28	2	ga_13
24	23	28	2	ga_13
23	24	27	2	ga_15
23	24	25	2	ga_13
27	24	25	2	ga_15
24	25	26	2	ga_12
23	28	30	2	ga_19
23	28	29	2	ga_30
30	28	29	2	ga_33
28	30	31	2	ga_32
28	30	32	2	ga_31
31	30	32	2	ga_18
32	39	41	2	ga_19
32	39	40	2	ga_30
41	39	40	2	ga_33
39	41	42	2	ga_32
39	41	43	2	ga_31
42	41	43	2	ga_18

43	48	49	2	ga_30
43	48	50	2	ga_19
49	48	50	2	ga_33
48	50	51	2	ga_32
48	50	52	2	ga_31
51	50	52	2	ga_18
52	59	60	2	ga_30
52	59	61	2	ga_19
60	59	61	2	ga_33
59	61	62	2	ga_32
59	61	63	2	ga_31
62	61	63	2	ga_18
61	63	76	2	ga_13
61	63	64	2	ga_13
76	63	64	2	ga_13
63	64	65	2	ga_15
64	65	66	2	ga_27
64	65	68	2	ga_27
66	65	68	2	ga_27
65	66	67	2	ga_25
65	66	70	2	ga_27
67	66	70	2	ga_25
65	68	69	2	ga_25
65	68	72	2	ga_27
69	68	72	2	ga_25
66	70	71	2	ga_25
66	70	74	2	ga_27
71	70	74	2	ga_25
68	72	73	2	ga_25
68	72	74	2	ga_27
73	72	74	2	ga_25
70	74	72	2	ga_27
70	74	75	2	ga_25
72	74	75	2	ga_25
63	76	77	2	ga_30
63	76	78	2	ga_19
77	76	78	2	ga_33
76	78	79	2	ga_32
76	78	80	2	ga_31
79	78	80	2	ga_18
78	80	85	2	ga_13
78	80	81	2	ga_13
85	80	81	2	ga_13
80	81	82	2	ga_15
81	82	83	2	ga_15

```
81    82    84     2    ga_15
83    82    84     2    ga_15
80    85    87     2    ga_19
80    85    86     2    ga_30
87    85    86     2    ga_33
85    87    88     2    ga_32
85    87    89     2    ga_31
88    87    89     2    ga_18
89    96    98     2    ga_19
89    96    97     2    ga_30
98    96    97     2    ga_33
96    98   100     2    ga_31
96    98    99     2    ga_32
100    98    99     2    ga_18
100   107   109     2    ga_19
100   107   108     2    ga_30
109   107   108     2    ga_33
107   109   110     2    ga_32
107   109   111     2    ga_31
110   109   111     2    ga_18
109   111   116     2    ga_13
109   111   112     2    ga_13
116   111   112     2    ga_13
111   112   113     2    ga_13
111   112   115     2    ga_15
113   112   115     2    ga_15
112   113   114     2    ga_12
46   116   111     2    ga_19
46   116   117     2    ga_33
111   116   117     2    ga_30

[ dihedrals ]
2     1     3     4          UNMAPPED
2     1    10    11          UNMAPPED
1     3     4     5          UNMAPPED
1    10    12    13          UNMAPPED
10    12    13    14          UNMAPPED
10    12    19    20          UNMAPPED
12    13    14    15          UNMAPPED
13    14    15    16          UNMAPPED
28    30    32    39          UNMAPPED
30    32    39    41          UNMAPPED
30    32    33    34          UNMAPPED
32    33    34    35          UNMAPPED
33    34    35    37          UNMAPPED
```

39	41	43	44		UNMAPPED
41	43	44	45		UNMAPPED
41	43	48	49		UNMAPPED
43	44	45	46		UNMAPPED
44	45	46	47		UNMAPPED
45	46	116	111		UNMAPPED
48	50	52	53		UNMAPPED
50	52	53	54		UNMAPPED
50	52	59	60		UNMAPPED
52	53	54	55		UNMAPPED
53	54	55	56		UNMAPPED
85	87	89	90		UNMAPPED
87	89	90	91		UNMAPPED
87	89	96	98		UNMAPPED
89	90	91	92		UNMAPPED
90	91	92	93		UNMAPPED
96	98	100	107		UNMAPPED
98	100	107	109		UNMAPPED
98	100	101	102		UNMAPPED
100	101	102	103		UNMAPPED
101	102	103	104		UNMAPPED
3	4	5	6	1	gd_34
4	5	6	7	1	gd_34
5	6	7	8	1	gd_34
6	7	8	9	1	gd_34
12	19	21	23	1	gd_14
19	21	23	28	1	gd_43
19	21	23	28	1	gd_44
21	23	24	25	1	gd_34
21	23	28	30	1	gd_42
21	23	28	30	1	gd_45
23	24	25	26	1	gd_23
23	28	30	32	1	gd_14
32	39	41	43	1	gd_14
43	48	50	52	1	gd_14
52	59	61	63	1	gd_14
59	61	63	76	1	gd_43
59	61	63	76	1	gd_44
61	63	76	78	1	gd_42
61	63	76	78	1	gd_45
61	63	64	65	1	gd_34
63	64	65	66	1	gd_40
63	64	65	68	1	gd_40
68	65	66	70	2	gi_1
66	65	68	72	2	gi_1

65	66	70	74	2	gi_1
65	68	72	74	2	gi_1
66	70	74	72	2	gi_1
68	72	74	70	2	gi_1
63	76	78	80	1	gd_14
76	78	80	85	1	gd_43
76	78	80	85	1	gd_44
78	80	85	87	1	gd_42
78	80	85	87	1	gd_45
78	80	81	82	1	gd_34
80	81	82	83	1	gd_34
80	81	82	84	1	gd_34
80	85	87	89	1	gd_14
89	96	98	100	1	gd_14
100	107	109	111	1	gd_14
107	109	111	116	1	gd_43
107	109	111	116	1	gd_44
109	111	116	46	1	gd_42
109	111	116	46	1	gd_45
109	111	112	113	1	gd_34
111	112	113	114	1	gd_23
85	80	87	86	2	gi_1
81	83	84	82	2	gi_2
80	78	85	81	2	gi_2
78	76	80	79	2	gi_1
81	84	83	82	2	gi_2
28	23	30	29	2	gi_1
23	27	25	24	2	gi_2
23	21	28	24	2	gi_2
21	19	23	22	2	gi_1
116	111	46	117	2	gi_1
111	115	113	112	2	gi_2
111	109	116	112	2	gi_2
109	107	111	110	2	gi_1
76	63	78	77	2	gi_1
74	70	72	75	2	gi_1
72	74	68	73	2	gi_1
70	74	66	71	2	gi_1
68	65	72	69	2	gi_1
66	65	70	67	2	gi_1
64	68	66	65	2	gi_1
63	61	76	64	2	gi_2
61	59	63	62	2	gi_1
74	72	70	75	2	gi_1
64	66	68	65	2	gi_1

```
19    12    21    20     2    gi_1
30    28    32    31     2    gi_1
41    39    43    42     2    gi_1
39    32    41    40     2    gi_1
50    48    52    51     2    gi_1
48    43    50    49     2    gi_1
59    52    61    60     2    gi_1
87    85    89    88     2    gi_1
98    96   100    99     2    gi_1
96    89    98    97     2    gi_1
107   100   109   108     2    gi_1
```

2.3.2 Automatic

Parameters obtained for Polymyxin B3 using first the manually specified Athenaeum, then the automatically generated Athenaeum.

```
[ atoms ]
1    %%%     1     TEST       C     1     0.00000   12.0110  ; UNMAPPED
2    %%%     1     TEST       O     2     0.00000   15.9994  ; UNMAPPED
3    %%%     1     TEST      CA     3     0.00000   14.0269  ; UNMAPPED
4    CH2     1     TEST      CB     4     0.00000   14.0269
5    CH2     1     TEST      CC     5     0.00000   14.0269
6    CH2     1     TEST      CD     6     0.00000   14.0269
7    CH2     1     TEST      CE     7     0.00000   14.0269
8    CH2     1     TEST      CG     8     0.00000   14.0269
9    CH3     1     TEST      CH     9     0.00000   15.0349
10       N     1     TEST       N    10    -0.31000   14.0067
11       H     1     TEST       H    11     0.31000    1.0080
12     CH1     1     TEST      CA    12     0.00000   13.0190
13     CH2     1     TEST      CB    13     0.00000   14.0269
14     CH2     1     TEST      CG    14     0.12700   14.0269
15      NL     1     TEST      ND    15     0.12900   14.0067
16       H     1     TEST     HD1    16     0.24800    1.0080
17       H     1     TEST     HD2    17     0.24800    1.0080
18       H     1     TEST     HD3    18     0.24800    1.0080
19       C     1     TEST       C    19     0.45000   12.0110
20       O     1     TEST       O    20    -0.45000   15.9994
21       N     1     TEST       N    21    -0.31000   14.0067
22       H     1     TEST       H    22     0.31000    1.0080
23     CH1     1     TEST      CA    23     0.00000   13.0190
24     CH1     1     TEST      CB    24     0.26600   13.0190
25      OA     1     TEST     OG1    25    -0.67400   15.9994
26       H     1     TEST     HG1    26     0.40800    1.0080
27     CH3     1     TEST     CG2    27     0.00000   15.0349
28       C     1     TEST       C    28     0.45000   12.0110
```

29	O	1	TEST	O	29	-0.45000	15.9994
30	N	1	TEST	N	30	-0.31000	14.0067
31	H	1	TEST	H	31	0.31000	1.0080
32	CH1	1	TEST	CA	32	0.00000	13.0190
33	CH2	1	TEST	CB	33	0.00000	14.0269
34	CH2	1	TEST	CG	34	0.12700	14.0269
35	NL	1	TEST	ND	35	0.12900	14.0067
36	H	1	TEST	HD1	36	0.24800	1.0080
37	H	1	TEST	HD2	37	0.24800	1.0080
38	H	1	TEST	HD3	38	0.24800	1.0080
39	C	1	TEST	C	39	0.45000	12.0110
40	O	1	TEST	O	40	-0.45000	15.9994
41	N	1	TEST	N	41	-0.31000	14.0067
42	H	1	TEST	H	42	0.31000	1.0080
43	CH1	1	TEST	CA	43	0.00000	13.0190
44	CH2	1	TEST	CB	44	0.00000	14.0269
45	CH2	1	TEST	CG	45	0.09000	14.0269
46	NE	1	TEST	ND	46	-0.11000	14.0067
47	H	1	TEST	HD1	47	0.24000	1.0080
48	C	1	TEST	C	48	0.45000	12.0110
49	O	1	TEST	O	49	-0.45000	15.9994
50	N	1	TEST	N	50	-0.31000	14.0067
51	H	1	TEST	H	51	0.31000	1.0080
52	CH1	1	TEST	CA	52	0.00000	13.0190
53	CH2	1	TEST	CB	53	0.00000	14.0269
54	CH2	1	TEST	CG	54	0.12700	14.0269
55	NL	1	TEST	ND	55	0.12900	14.0067
56	H	1	TEST	HD1	56	0.24800	1.0080
57	H	1	TEST	HD2	57	0.24800	1.0080
58	H	1	TEST	HD3	58	0.24800	1.0080
59	C	1	TEST	C	59	0.45000	12.0110
60	O	1	TEST	O	60	-0.45000	15.9994
61	N	1	TEST	N	61	-0.31000	14.0067
62	H	1	TEST	H	62	0.31000	1.0080
63	CH1	1	TEST	CA	63	0.00000	13.0190
64	CH2	1	TEST	CB	64	0.00000	14.0269
65	C	1	TEST	CG	65	0.00000	12.0110
66	C	1	TEST	CD1	66	-0.14000	12.0110
67	HC	1	TEST	HD1	67	0.14000	1.0080
68	C	1	TEST	CD2	68	-0.14000	12.0110
69	HC	1	TEST	HD2	69	0.14000	1.0080
70	C	1	TEST	CE1	70	-0.14000	12.0110
71	HC	1	TEST	HE1	71	0.14000	1.0080
72	C	1	TEST	CE2	72	-0.14000	12.0110
73	HC	1	TEST	HE2	73	0.14000	1.0080

74	C	1	TEST	CZ	74	-0.14000	12.0110
75	HC	1	TEST	HZ	75	0.14000	1.0080
76	C	1	TEST	C	76	0.45000	12.0110
77	O	1	TEST	O	77	-0.45000	15.9994
78	N	1	TEST	N	78	-0.31000	14.0067
79	H	1	TEST	H	79	0.31000	1.0080
80	CH1	1	TEST	CA	80	0.00000	13.0190
81	CH2	1	TEST	CB	81	0.00000	14.0269
82	CH1	1	TEST	CG	82	0.00000	13.0190
83	CH3	1	TEST	CD1	83	0.00000	15.0349
84	CH3	1	TEST	CD2	84	0.00000	15.0349
85	C	1	TEST	C	85	0.45000	12.0110
86	O	1	TEST	O	86	-0.45000	15.9994
87	N	1	TEST	N	87	-0.31000	14.0067
88	H	1	TEST	H	88	0.31000	1.0080
89	CH1	1	TEST	CA	89	0.00000	13.0190
90	CH2	1	TEST	CB	90	0.00000	14.0269
91	CH2	1	TEST	CG	91	0.12700	14.0269
92	NL	1	TEST	ND	92	0.12900	14.0067
93	H	1	TEST	HD1	93	0.24800	1.0080
94	H	1	TEST	HD2	94	0.24800	1.0080
95	H	1	TEST	HD3	95	0.24800	1.0080
96	C	1	TEST	C	96	0.45000	12.0110
97	O	1	TEST	O	97	-0.45000	15.9994
98	N	1	TEST	N	98	-0.31000	14.0067
99	H	1	TEST	H	99	0.31000	1.0080
100	CH1	1	TEST	CA	100	0.00000	13.0190
101	CH2	1	TEST	CB	101	0.00000	14.0269
102	CH2	1	TEST	CG	102	0.12700	14.0269
103	NL	1	TEST	ND	103	0.12900	14.0067
104	H	1	TEST	HD1	104	0.24800	1.0080
105	H	1	TEST	HD2	105	0.24800	1.0080
106	H	1	TEST	HD3	106	0.24800	1.0080
107	C	1	TEST	C	107	0.45000	12.0110
108	O	1	TEST	O	108	-0.45000	15.9994
109	N	1	TEST	N	109	-0.31000	14.0067
110	H	1	TEST	H	110	0.31000	1.0080
111	CH1	1	TEST	CA	111	0.00000	13.0190
112	CH1	1	TEST	CB	112	0.26600	13.0190
113	OA	1	TEST	OG1	113	-0.67400	15.9994
114	H	1	TEST	HG1	114	0.40800	1.0080
115	CH3	1	TEST	CG2	115	0.00000	15.0349
116	C	1	TEST	C	116	0.45000	12.0110
117	O	1	TEST	O	117	-0.45000	15.9994

```
[ bonds ]
1      2      2     gb_UNMAPPED
1      3      2     gb_UNMAPPED
1     10      2     gb_10
3      4      2     gb_27
4      5      2     gb_27
5      6      2     gb_27
6      7      2     gb_27
7      8      2     gb_27
8      9      2     gb_27
10     11      2     gb_2
10     12      2     gb_21
12     13      2     gb_27
12     19      2     gb_27
13     14      2     gb_27
14     15      2     gb_21
15     16      2     gb_2
15     17      2     gb_2
15     18      2     gb_2
19     20      2     gb_5
19     21      2     gb_10
21     22      2     gb_2
21     23      2     gb_21
23     24      2     gb_27
23     28      2     gb_27
24     27      2     gb_27
24     25      2     gb_18
25     26      2     gb_1
28     30      2     gb_10
28     29      2     gb_5
30     31      2     gb_2
30     32      2     gb_21
32     39      2     gb_27
32     33      2     gb_27
33     34      2     gb_27
34     35      2     gb_21
35     37      2     gb_2
35     36      2     gb_2
35     38      2     gb_2
39     41      2     gb_10
39     40      2     gb_5
41     42      2     gb_2
41     43      2     gb_21
43     44      2     gb_27
43     48      2     gb_27
```

44	45	2	gb_27
45	46	2	gb_21
46	47	2	gb_2
46	116	2	gb_10
48	49	2	gb_5
48	50	2	gb_10
50	51	2	gb_2
50	52	2	gb_21
52	53	2	gb_27
52	59	2	gb_27
53	54	2	gb_27
54	55	2	gb_21
55	56	2	gb_2
55	57	2	gb_2
55	58	2	gb_2
59	60	2	gb_5
59	61	2	gb_10
61	62	2	gb_2
61	63	2	gb_21
63	76	2	gb_27
63	64	2	gb_27
64	65	2	gb_27
65	66	2	gb_16
65	68	2	gb_16
66	67	2	gb_3
66	70	2	gb_16
68	69	2	gb_3
68	72	2	gb_16
70	71	2	gb_3
70	74	2	gb_16
72	73	2	gb_3
72	74	2	gb_16
74	75	2	gb_3
76	77	2	gb_5
76	78	2	gb_10
78	79	2	gb_2
78	80	2	gb_21
80	85	2	gb_27
80	81	2	gb_27
81	82	2	gb_27
82	83	2	gb_27
82	84	2	gb_27
85	87	2	gb_10
85	86	2	gb_5
87	88	2	gb_2

```
87    89     2    gb_21
89    90     2    gb_27
89    96     2    gb_27
90    91     2    gb_27
91    92     2    gb_21
92    93     2    gb_2
92    94     2    gb_2
92    95     2    gb_2
96    98     2    gb_10
96    97     2    gb_5
98   100     2    gb_21
98    99     2    gb_2
100   107     2    gb_27
100   101     2    gb_27
101   102     2    gb_27
102   103     2    gb_21
103   104     2    gb_2
103   105     2    gb_2
103   106     2    gb_2
107   109     2    gb_10
107   108     2    gb_5
109   110     2    gb_2
109   111     2    gb_21
111   116     2    gb_27
111   112     2    gb_27
112   113     2    gb_18
112   115     2    gb_27
113   114     2    gb_1
116   117     2    gb_5

[ angles ]
2     1     3     2    ga_UNMAPPED
2     1    10     2    ga_UNMAPPED
3     1    10     2    ga_UNMAPPED
1     3     4     2    ga_UNMAPPED
3     4     5     2    ga_15
4     5     6     2    ga_15
5     6     7     2    ga_15
6     7     8     2    ga_15
7     8     9     2    ga_15
1    10    11     2    ga_32
1    10    12     2    ga_31
11    10    12     2    ga_18
10    12    13     2    ga_13
10    12    19     2    ga_13
```

13	12	19	2	ga_13
12	13	14	2	ga_15
13	14	15	2	ga_15
14	15	16	2	ga_11
14	15	17	2	ga_11
14	15	18	2	ga_11
16	15	17	2	ga_10
16	15	18	2	ga_10
17	15	18	2	ga_10
12	19	20	2	ga_30
12	19	21	2	ga_19
20	19	21	2	ga_33
19	21	22	2	ga_32
19	21	23	2	ga_31
22	21	23	2	ga_18
21	23	24	2	ga_13
21	23	28	2	ga_13
24	23	28	2	ga_13
23	24	27	2	ga_15
23	24	25	2	ga_13
27	24	25	2	ga_15
24	25	26	2	ga_12
23	28	30	2	ga_19
23	28	29	2	ga_30
30	28	29	2	ga_33
28	30	31	2	ga_32
28	30	32	2	ga_31
31	30	32	2	ga_18
30	32	39	2	ga_13
30	32	33	2	ga_13
39	32	33	2	ga_13
32	33	34	2	ga_15
33	34	35	2	ga_15
34	35	37	2	ga_11
34	35	36	2	ga_11
34	35	38	2	ga_11
37	35	36	2	ga_10
37	35	38	2	ga_10
36	35	38	2	ga_10
32	39	41	2	ga_19
32	39	40	2	ga_30
41	39	40	2	ga_33
39	41	42	2	ga_32
39	41	43	2	ga_31
42	41	43	2	ga_18

41	43	44	2	ga_13
41	43	48	2	ga_13
44	43	48	2	ga_13
43	44	45	2	ga_15
44	45	46	2	ga_13
45	46	47	2	ga_20
45	46	116	2	ga_33
47	46	116	2	ga_23
43	48	49	2	ga_30
43	48	50	2	ga_19
49	48	50	2	ga_33
48	50	51	2	ga_32
48	50	52	2	ga_31
51	50	52	2	ga_18
50	52	53	2	ga_13
50	52	59	2	ga_13
53	52	59	2	ga_13
52	53	54	2	ga_15
53	54	55	2	ga_15
54	55	56	2	ga_11
54	55	57	2	ga_11
54	55	58	2	ga_11
56	55	57	2	ga_10
56	55	58	2	ga_10
57	55	58	2	ga_10
52	59	60	2	ga_30
52	59	61	2	ga_19
60	59	61	2	ga_33
59	61	62	2	ga_32
59	61	63	2	ga_31
62	61	63	2	ga_18
61	63	76	2	ga_13
61	63	64	2	ga_13
76	63	64	2	ga_13
63	64	65	2	ga_15
64	65	66	2	ga_27
64	65	68	2	ga_27
66	65	68	2	ga_27
65	66	67	2	ga_25
65	66	70	2	ga_27
67	66	70	2	ga_25
65	68	69	2	ga_25
65	68	72	2	ga_27
69	68	72	2	ga_25
66	70	71	2	ga_25

66	70	74	2	ga_27
71	70	74	2	ga_25
68	72	73	2	ga_25
68	72	74	2	ga_27
73	72	74	2	ga_25
70	74	72	2	ga_27
70	74	75	2	ga_25
72	74	75	2	ga_25
63	76	77	2	ga_30
63	76	78	2	ga_19
77	76	78	2	ga_33
76	78	79	2	ga_32
76	78	80	2	ga_31
79	78	80	2	ga_18
78	80	85	2	ga_13
78	80	81	2	ga_13
85	80	81	2	ga_13
80	81	82	2	ga_15
81	82	83	2	ga_15
81	82	84	2	ga_15
83	82	84	2	ga_15
80	85	87	2	ga_19
80	85	86	2	ga_30
87	85	86	2	ga_33
85	87	88	2	ga_32
85	87	89	2	ga_31
88	87	89	2	ga_18
87	89	90	2	ga_13
87	89	96	2	ga_13
90	89	96	2	ga_13
89	90	91	2	ga_15
90	91	92	2	ga_15
91	92	93	2	ga_11
91	92	94	2	ga_11
91	92	95	2	ga_11
93	92	94	2	ga_10
93	92	95	2	ga_10
94	92	95	2	ga_10
89	96	98	2	ga_19
89	96	97	2	ga_30
98	96	97	2	ga_33
96	98	100	2	ga_31
96	98	99	2	ga_32
100	98	99	2	ga_18
98	100	107	2	ga_13

```
98   100   101     2    ga_13
107   100   101     2    ga_13
100   101   102     2    ga_15
101   102   103     2    ga_15
102   103   104     2    ga_11
102   103   105     2    ga_11
102   103   106     2    ga_11
104   103   105     2    ga_10
104   103   106     2    ga_10
105   103   106     2    ga_10
100   107   109     2    ga_19
100   107   108     2    ga_30
109   107   108     2    ga_33
107   109   110     2    ga_32
107   109   111     2    ga_31
110   109   111     2    ga_18
109   111   116     2    ga_13
109   111   112     2    ga_13
116   111   112     2    ga_13
111   112   113     2    ga_13
111   112   115     2    ga_15
113   112   115     2    ga_15
112   113   114     2    ga_12
46   116   111     2    ga_19
46   116   117     2    ga_33
111   116   117     2    ga_30

[ dihedrals ]
2     1     3     4          UNMAPPED
2     1    10    11          UNMAPPED
1     3     4     5          UNMAPPED
12    13    14    15          UNMAPPED
32    33    34    35          UNMAPPED
43    44    45    46          UNMAPPED
45    46   116   111          UNMAPPED
52    53    54    55          UNMAPPED
89    90    91    92          UNMAPPED
100   101   102   103          UNMAPPED
3     4     5     6     1    gd_34
4     5     6     7     1    gd_34
5     6     7     8     1    gd_34
6     7     8     9     1    gd_34
1    10    12    19     1    gd_43
1    10    12    19     1    gd_44
10    12    13    14     1    gd_34
```

10	12	19	21	1	gd_42
10	12	19	21	1	gd_45
13	14	15	16	1	gd_29
12	19	21	23	1	gd_14
19	21	23	28	1	gd_43
19	21	23	28	1	gd_44
21	23	24	25	1	gd_34
21	23	28	30	1	gd_42
21	23	28	30	1	gd_45
23	24	25	26	1	gd_23
23	28	30	32	1	gd_14
28	30	32	39	1	gd_43
28	30	32	39	1	gd_44
30	32	39	41	1	gd_42
30	32	39	41	1	gd_45
30	32	33	34	1	gd_34
33	34	35	37	1	gd_29
32	39	41	43	1	gd_14
39	41	43	48	1	gd_43
39	41	43	48	1	gd_44
41	43	44	45	1	gd_34
41	43	48	50	1	gd_42
41	43	48	50	1	gd_45
44	45	46	116	1	gd_39
43	48	50	52	1	gd_14
48	50	52	59	1	gd_43
48	50	52	59	1	gd_44
50	52	53	54	1	gd_34
50	52	59	61	1	gd_42
50	52	59	61	1	gd_45
53	54	55	56	1	gd_29
52	59	61	63	1	gd_14
59	61	63	76	1	gd_43
59	61	63	76	1	gd_44
61	63	76	78	1	gd_42
61	63	76	78	1	gd_45
61	63	64	65	1	gd_34
63	64	65	66	1	gd_40
63	64	65	68	1	gd_40
68	65	66	70	2	gi_1
66	65	68	72	2	gi_1
65	66	70	74	2	gi_1
65	68	72	74	2	gi_1
66	70	74	72	2	gi_1
68	72	74	70	2	gi_1

63	76	78	80	1	gd_14
76	78	80	85	1	gd_43
76	78	80	85	1	gd_44
78	80	85	87	1	gd_42
78	80	85	87	1	gd_45
78	80	81	82	1	gd_34
80	81	82	83	1	gd_34
80	81	82	84	1	gd_34
80	85	87	89	1	gd_14
85	87	89	96	1	gd_43
85	87	89	96	1	gd_44
87	89	90	91	1	gd_34
87	89	96	98	1	gd_42
87	89	96	98	1	gd_45
90	91	92	93	1	gd_29
89	96	98	100	1	gd_14
96	98	100	107	1	gd_43
96	98	100	107	1	gd_44
98	100	107	109	1	gd_42
98	100	107	109	1	gd_45
98	100	101	102	1	gd_34
101	102	103	104	1	gd_29
100	107	109	111	1	gd_14
107	109	111	116	1	gd_43
107	109	111	116	1	gd_44
109	111	116	46	1	gd_42
109	111	116	46	1	gd_45
109	111	112	113	1	gd_34
111	112	113	114	1	gd_23
21	19	23	22	2	gi_1
19	12	21	20	2	gi_1
30	28	32	31	2	gi_1
28	23	30	29	2	gi_1
41	39	43	42	2	gi_1
39	32	41	40	2	gi_1
50	48	52	51	2	gi_1
48	43	50	49	2	gi_1
61	59	63	62	2	gi_1
59	52	61	60	2	gi_1
78	76	80	79	2	gi_1
76	63	78	77	2	gi_1
87	85	89	88	2	gi_1
85	80	87	86	2	gi_1
99	100	96	98	2	gi_1
96	89	98	97	2	gi_1

109	107	111	110	2	gi_1
107	100	109	108	2	gi_1
81	83	84	82	2	gi_2
80	78	85	81	2	gi_2
81	84	83	82	2	gi_2
23	27	25	24	2	gi_2
23	21	28	24	2	gi_2
116	111	46	117	2	gi_1
111	115	113	112	2	gi_2
111	109	116	112	2	gi_2
74	70	72	75	2	gi_1
72	74	68	73	2	gi_1
70	74	66	71	2	gi_1
68	65	72	69	2	gi_1
66	65	70	67	2	gi_1
64	68	66	65	2	gi_1
63	61	76	64	2	gi_2
74	72	70	75	2	gi_1
64	66	68	65	2	gi_1
12	10	19	13	2	gi_2
10	1	12	11	2	gi_1
32	30	39	33	2	gi_2
43	41	48	44	2	gi_2
52	50	59	53	2	gi_2
89	87	96	90	2	gi_2
100	98	107	101	2	gi_2
46	45	116	47	2	gi_1
72	68	74	73	2	gi_1
70	66	74	71	2	gi_1
